# Efficiency of Lidocaine Intramuscular and Intraosseous Trigger Point Injections in the Treatment of Residual Chronic Pain after Degenerative Lumbar Spinal Stenosis Decompression Surgery

**DOI:** 10.3390/jcm13185437

**Published:** 2024-09-13

**Authors:** Mustafa Al-Zamil, Natalia G. Kulikova, Natalia A. Shnayder, Natalia B. Korchazhkina, Marina M. Petrova, Tatyana I. Mansur, Vasilissa V. Blinova, Zarina M. Babochkina, Ekaterina S. Vasilyeva, Ivan V. Zhhelambekov

**Affiliations:** 1 Department of Physiotherapy, Faculty of Continuing Medical Education, Peoples’ Friendship University of Russia, 117198 Moscow, Russia; kulikovang777@mail.ru (N.G.K.); vasilissablinova@yandex.ru (V.V.B.); 2 Department of Sports Medicine and Medical Rehabilitation, I.M. Sechenov First Moscow State Medical University, 119991 Moscow, Russia; 3 Institute of Personalized Psychiatry and Neurology, V.M. Bekhterev National Medical Research Centre for Psychiatry and Neurology, 192019 Saint Petersburg, Russia; 4 Shared Core Facilities “Molecular and Cell Technologies”, Professor V. F. Voino-Yasenetsky Krasnoyarsk State Medical University, 660022 Krasnoyarsk, Russia; stk99@yandex.ru; 5 Department of Restorative Medicine and Biomedical Technologies, Federal State Educational Institution of Higher Education, Moscow State Medical and Dental University Named after A.I. Evdokimov, Ministry of Health of Russia, 127473 Moscow, Russia; n9857678103@gmail.com (N.B.K.); e_vasilieva@inbox.ru (E.S.V.); 6 General Medical Practice Department, Medical Institute of PFUR, Peoples’ Friendship University of Russia, 117198 Moscow, Russia; mansur_ti@pfur.ru; 7 Department of Restorative Medicine and Neurorehabilitation, Medical Dental Institute, 127253 Moscow, Russia; zzangieva2008@yandex.ru (Z.M.B.); aiybolit69@mail.ru (I.V.Z.)

**Keywords:** paravertebral blockade, intraosseous blockade, lidocaine blockade, posterior superior iliac spine, gate control theory of pain, degenerative lumbar spinal stenosis decompression surgery, low back pain, trigger point injection

## Abstract

**Introduction**: Despite the long-term use of intramuscular and intraosseous lidocaine trigger point injections (LTPI) in the treatment of patients with low back pain, there have been no studies examining their efficiency in treatment of residual pain after degenerative lumbar spinal stenosis (DLSS) decompression surgery. The purpose of our research is to examine the LTPI efficiency in the treatment of residual lumbar pain after DLSS decompression surgery and to compare the analgesic and recovery effects of intramuscular and intraosseous LTPI administered in the L4–S1 region and in the posterior superior iliac spine (PSIS) after treatment and during four months of follow-up. **Materials and Methods**: We observed 99 patients (F:50, M:49) aged 42 to 59 years with residual neurological disorders after DLSS decompression surgery. In all patients, the pain syndrome exceeded 6 points on the VAS and averaged 7.2 ± 0.11 points. The control group (*n* = 21) underwent only pharmacotherapy. In addition to pharmacotherapy, the LTPI group underwent intramuscular LTPI in L4–S1 (*n* = 20), intramuscular LTPI in the PSIS (*n* = 19), intraosseous LTPI in L5, S1 (*n* = 20), and intraosseous LTPI in the PSIS (*n* = 19). A neurological examination was carried out before treatment, 7 days after completion of treatment, and at the end of the second and fourth months of the follow-up period. **Results**: In the control group, intramuscular LTPI in L4–S1 subgroup, intramuscular LTPI in PSIS subgroup, intraosseous LTPI in L5, S1 subgroup, and intraosseous LTPI in PSIS subgroup, the severity of pain decreased after treatment by 27.1% (*p* ≤ 0.05), 41.7% (*p* ≤ 0.01), 50.7% (*p* ≤ 0.01), 69% (*p* ≤ 0.01), and 84.7% (*p* ≤ 0.01), respectively, and at the end of the second month of follow-up, by 14.3% (*p* > 1), 29.2% (*p* ≤ 0.05), 38% (*p* ≤ 0.01), 53.5% (*p* ≤ 0.01), and 72.2% (*p* ≤ 0.01), respectively. Reduction of neurogenic claudication, regression of sensory deficit, increase of daily step activity, and improvement of quality of life after treatment were noted in intramuscular LTPI subgroups by 19.6% (*p* ≤ 0.05), 36.4 (*p* ≤ 0.05), 40.3% (*p* ≤ 0.01), and 21.0% (*p* ≤ 0.05), respectively, and in interosseous LTPI subgroups by 48.6% (*p* ≤ 0.01), 67.4% (*p* ≤ 0.01), 68.3% (*p* ≤ 0.01), and 46% (*p* ≤ 0.01), respectively. **Conclusions**: LTPI is highly effective in the treatment of patients with residual pain after DLSS decompression surgery. High analgesic effect, significant regression of sensory deficits and gait disorders, and remarkable improvement of daily step activity and quality of life are noted not only after the end of LTPI treatment but also continue for at least 2 months after treatment. Intraosseous LTPI is more effective than intramuscular LTPI by 92%, and LTPI in PSIS is more effective than LTPI in L4–S1 by 28.6%.

## 1. Introduction

Degenerative lumbar spinal stenosis (DLSS) was first described 124 years ago by Sachs B and Fraenkel J [1]. DLSS is a common cause of disabling back and lower extremity pain in older adults and is responsible for pain in 50% of patients with low back pain [2]. This pathological condition usually develops as a result of degeneration of the intervertebral discs and facet joints, which leads to narrowing of the spinal canal and neural foramina compressing the nerves traveling through the lower back into the legs [3,4,5,6]. The relevance of this problem lies in the fact that the disease occurs in 31.5% of the working population aged 45 to 64 years [7,8]. In almost all patients, they may experience back and leg pain, progressive neurological deficits, bowel and bladder dysfunction, and neurogenic intermittent claudication (pseudoclaudication) [9]. Due to limited physical activity and chronic pain, a deterioration in quality of life may develop [10]. 

In symptomatic spinal stenosis, surgical treatment maintains substantially greater clinical improvement than nonsurgical treatment [11]. However, the literature has not found statistically significant differences between the clinical outcomes of laminectomy and laminotomy [12]. On the other hand, posterior fixation procedures with facet distraction without decompression have recently been shown to produce good clinical results in 87% of patients [13].

Nevertheless, residual leg pain and positive sensory symptoms were diagnosed in 30.3% of patients for one year and more after decompression of lumbar spinal stenosis [14]. The most common causes of persistent pain and neurological deficit after DLSS decompression are epidural fibrosis [15], re-herniation after discectomy [16], spinal fusion failure [17], post-spinal fusion “adjacent segment disease” [18], damage to the nerve fibers prior to surgery or during the surgical process [19], and postoperative lumboparaspinal compartment syndrome [20]. Unfortunately, in many cases, pharmacotherapy alone cannot relieve pain and reduce neurological disorders [21]. Moreover, repeated surgical treatment may not always provide a satisfactory analgesic and functional recovery effect [22]. However, patients with pain that persists to unbearable levels do not always find relief with NSAIDs or physical therapy [23]. 

Lidocaine trigger point injection (LTPI) is often used as an intractable pain management method for DLSS [24,25,26]. The mechanism of action of these procedures is not entirely clear. Lidocaine’s effect is due to blocking sodium channels on the inner surface of nerve cell membranes, keeping them open, which prevents nerve depolarization. Thus, lidocaine not only prevents the transmission of the action potential but also prevents the depolarization process [27]. The analgesic effect of intramuscular injection of 2%–4 mL lidocaine can last a maximum of 8 h with a radius of influence of no more than 2 cm [28]. Such a short and limited effect does not explain the clear reduction in pain sensory deficits and gait disturbances within 24 h and sometimes several days after LTPI in the treatment of patients with severe low back pain. In this regard, there is reason to suspect that LTPI is not only local but also has segmental and suprasegmental mechanisms [29,30]. The segmental mechanism is explained by the gate control theory of pain, blocking slow afferents (unmyelinated fibers) that have an inhibitory effect on the gelatinous substance of the posterior horn of the spinal cord, which serves as an inhibitory interneuron for nociceptive afferents [31,32]. As a result of this action, pain segmentally decreases with normalization of reflex increased muscle tone in the paravertebral, gluteal, and piriformis muscles [33]. Moreover, trigger points often coincide with acupuncture points. In such cases, the effect of lidocaine on ergoreceptors has a parasegmental analgesic effect due to the release of central endorphins [23,29,34]. 

Trigger points in patients with residual neurological disorders after DLSS decompression surgery are heterogeneous in structure and characteristics and contain one or more sensitized nociceptive nerve endings [28]. Intramuscular ones consist of a dense group of skeletal muscles (localized contraction nodes) [35]. Ligamentous trigger points consist of stretched or inflamed ligaments [36]. Periosteal trigger points form from inflamed and overstretched tendons where muscles and tendons attach to bones [37]. While intraosseous trigger points are little studied points in the bones. These hyperirritable areas are typically tender to pressure and can cause characteristic referred pain and reflexogenic muscle hypertonicity, motor dysfunction, vasospasm, and autonomic phenomena [25].

Some studies have noted that intraosseous injection of lidocaine into trigger points has a more pronounced effect than intramuscular administration [38]. In Russia, this method is called intraosseous blockade. Intraosseous LTPI is an intraosseous injection technique whereby a local anesthetic is injected into the cancellous bone at the site of pain [39]. It is difficult to determine from the literature where interosseous LTPI was first performed. However, the first description of this method was made in 1918 by a dentist from San Francisco [40]. G.M. Shulyak G.M. (1898–1967) was the first to use intraosseous LTPI as a method of treating low back pain in patients with lumbar osteochondrosis. The results of his work were published in his monograph “Intraosseous method of pain relief and its anatomical basis” in 1953 [23]. If Yankovsky made a great contribution to the experimental development of the physiology and pathophysiology of intraosseous receptors with the discovery of new possibilities for intraosteotherapy, then Sokov’s work in 1986 turned out to be the clinical translation of these achievements in clinical practice, especially in the treatment of vertebrogenic and neuropathic pain [41,42,43]. According to the authors of the technique, the high efficiency of intraosseous LTPI lies in the presence of a large number of slow afferents in the cancellous bone tissue [44]. 

Previous studies have found that pain from low back pain is more likely to radiate to the posterior superior iliac spine (PSIS) than to the lumbosacral region. In this regard, intramuscular and intraosseous LTPI are performed not only in the area of pain in the lumbosacral region but also in the PSIS projection [5,6].

Despite the long-term use of intramuscular and intraosseous LTPI in the treatment of patients with low back pain, there have been no studies examining their efficiency in the treatment of residual pain after DLSS decompression surgery. Moreover, the comparative analysis of intramuscular and intraosseous LTPI remains poorly studied.

The purpose of our research is to examine the LTPI efficiency in the treatment of residual lumbar pain after DLSS decompression surgery and to compare the analgesic and recovery effects of intramuscular and intraosseous LTPI administered in the L4–S1 region and in the posterior superior iliac spine (PSIS) after treatment and during four months of follow-up.

## 2. Materials and Methods

### 2.1. Study Design and Population 

In this randomized single-center clinical trial, we observed 224 patients with persisting severe lumbar region pain and neurogenic claudication after lumbar spine decompression surgery (postlaminectomy syndrome, not elsewhere classified), ICD-10-CM code: M96.1 [45].

#### 2.1.1. Inclusion and Exclusion Criteria

Inclusion criteria in the study:

1. european;
2. adult men and women from 40 to 60 years old;
3. residual pain syndrome is older than 6 months but less than 3 years after DLSS decompression surgery;
4. localization of the maximum pain syndrome in the lumbosacral joint;
5. the severity of residual pain on the visual analog scale (VAS) is 6 scores and higher;
6. DLSS decompression surgery was completed without complications and without significant negative dynamics, according to magnetic resonance imaging (MRI) and electroneuromyography (ENMG) data.;
7. signed voluntary informed consent to participate in this study.

Exclusion criteria in the study:

1. presence of allergic reactions to any of the drugs used
2. severely cognitive disorders;
3. foraminal and lateral location of spinal canal stenosis and severe narrowing on MRI;
4. distal polyneuropathy of the peroneal and tibial nerves according to electroneuromyography;
5. ankylosing spondylitis;
6. rheumatoid diseases;
7. atherosclerotic peripheral arterial disease of the lower extremities;
8. muscular dystrophies of the lower extremities;
9. motor deficit;
10. bladder and bowel dysfunction;
11. diabetes mellitus;
12. pregnancy;
13. undergoing physiotherapy or acupuncture treatment.

#### 2.1.2. Informed Consent and Approval of the Local Medical Ethics Committee

After an explanation of the medical condition, the purpose and benefits of the test, procedure, or treatment, and a description of the proposed test, procedure, or treatment, including possible complications or adverse events, all patients signed a voluntary Informed Consent in Research and Clinical Care. 

The study protocol was approved by the local medical ethical committee of the Peoples’ Friendship University of Russia (protocol No. 130, 5 December 2022). All procedures were in accordance with the 1984 Declaration of Helsinki and its subsequent amendments. All patients read the above article in its entirety, including text, figures, and supplementary material, and consented to its publication.

There is no compensation for participation in this study. The researchers were not compensated for their work. The study was conducted within the framework of the research program of the Department of Physiotherapy, Faculty of Continuing Medical Education, the Peoples’ Friendship University of Russia.

#### 2.1.3. Distribution of Patients in Groups

The study included 224 patients (114 women, 110 men) with severe low back pain that persisted after DLSS decompression surgery. 112 of these patients were excluded. This is because 52 patients did not meet the inclusion criteria, and 60 patients refused further participation.

112 patients (female—57, male—55) meeting all inclusion criteria were randomized in a 1:1:1:1:1 ratio. 9 patients discontinued participation due to withdrawal of consent, loss of follow-up, and allergic reaction to one of the used drugs. 

**Figure 1:**
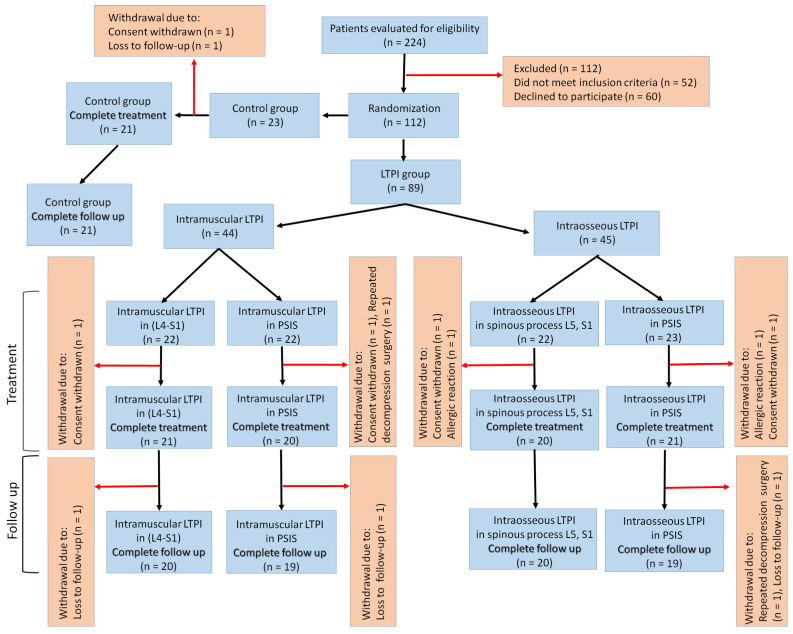
Flow chart of study population selection. Notes: LTPI: Lidocaine trigger point injection; L4–S1—parvertebral space between the fourth lumbar and first sacral vertebrae; PSIS—posterior superior iliac spine; S1—first sacral vertebra; L5—fifth lumbar vertebra.

As a result, the number of patients completing treatment was reduced to 103 patients. (female—53, male—50). However, during the follow-up period, four patients were excluded because three patients were lost to follow-up and one patient underwent repeated decompression surgery. Thus, 99 patients (F: 50, M: 49) were followed up for 4 months after treatment. The control group included 21 patients who received only standard pharmacotherapy. Half of the LTPI group additional to standard pharmacotherapy underwent intramuscular LTPI in PSIS (*n* = 19) and in the paravertebral L4–S1 region (*n* = 20) on the side of the pain syndrome. The second half, in addition to standard pharmacotherapy, completed the course of intraosseous LTPI in PSIS (*n* = 19), and 20 patients after bilateral DLSS decompression via unilateral laminotomy underwent intraosseous LTPI in the spinous process of L5 or S1 (Figure 1). 

#### 2.1.4. Demographic and Clinical Characteristics of the Participants

Demographic data and clinical characteristics of the patients are summarized in Table 1. The age of the patients ranged from 42 to 59 years and averaged 53.4 ± 4.98 years. The male-to-female ratio of the study participants, control group, and all LTPI subgroups was 1:1. In all patients, the duration of low back pain ranged from 6 to 35 months and averaged 21.6 ± 7.84 months. On a 10-point visual analogue scale, the severity of low back pain was assessed from 6 to 10 points with an average score of 7.82 ± 1.20 points. Between groups differences in patient`s number, age, gender ratio, disease duration after DLSS decompression surgery, and severity of low back pain assessed by VAS were not statistically significant (*p*-value > 0.05).

### 2.2. Sample Size Calculation

To determine the minimum number of subjects in each group, we used the sample size calculator at https://clincalc.com/stats/samplesize.aspx site (accessed on 5 January 2023). A literature search revealed that a previous study compared the effectiveness of intraosseous blockade and intramuscular blockade in the treatment of patients with severe low back pain [38]. The results of this study showed that the severity of pain according to VAS (M ± SD) decreased after the use of intraosseous blockade from 7.9 ± 1.1 points to 2.1 ± 1.3 points and was lower than after interstitial blockade by 53.3%. Therefore, according to the sample size calculation, the minimum number of patients in each group with a power value of 95%, the probability of a type-I error of 0.01, and the expected significance level (*p*-value) of 0.05 should be 9 patients or more. 

### 2.3. Clinical Examinations and Diagnostics 

A neurological examination was performed by a board-certified neurologist blinded to participant status. Neurological data were recorded in a standardized form, indicating the presence or absence of abnormalities. Anamnesis and physical and laboratory examination were studied in detail. Mental pathology, cranial nerve abnormalities, cerebellar and gait disorders, motor deficits, changes in reflexes, development of pathological reflexes, and the presence of negative and positive sensory symptoms were examined. A neurological examination was carried out before treatment, 7 days after completion of treatment, and at the end of the second and fourth months of the follow-up period. 

#### 2.3.1. Pain Assessment 

The visual analog scale (VAS) has long been used to assess pain for clinical and research purposes. It was first used in 1921 by Hayes and Patterson. A simple method is based on determining the subjective pain syndrome along a vertical or horizontal line consisting of 11 points with even distances, which corresponds to an 11-point scale from 0 (no pain) to 10 (unbearable pain). VAS is widely used due to its simplicity, rapidity of determination, and ability to adapt to a wide range of populations and settings. Except that the VAS is more sensitive to small changes than simple descriptive ordinal scales that rate symptoms, for example, as mild or mild, moderate or severe, or intolerable.

#### 2.3.2. Assessment of Impaired Tactile Sensation in L5 and S1 Dermatomes 

Tactile sensation was assessed in L5 and S1 dermatomes compared with tactile sensitivity on the thigh. Patients objectively assessed the tactile sensation of the L5 and S1 dermatomes of the foot and lower leg on a 5-point scale in comparison with sensation at the level of the anterior thigh. The evaluation used a Touch Test filament of 6.65 g. When the score is 0, there is no sensation; with a score of 5, tactile sensation is normal.

#### 2.3.3. Assessment of Neurogenic Claudication

Neurogenic claudication was assessed using the Zurich Claudication Questionnaire (ZCQ). ZCQ is a dedicated DLSS assessment tool. Many studies have demonstrated the accuracy, specificity, validity, and reliability of the ZCQ in measuring neurogenic claudication and walking capacity in patients with DLSS. This questionnaire measures symptoms and limitations in physical activity within the prior month and includes 12 questions related to three components (scales). The first scale is designed to measure the severity of symptoms, and the second scale is designed to measure functional disability caused by spinal stenosis [46].

The first scale (symptom severity) encompasses seven questions related to back and lower extremity symptoms such as pain, numbness, weakness, and imbalance. There are two domains: pain (questions 1–4) and neuroischemic domain (questions 5–7). Each of the seven questions is rated on a scale of 1 to 5, with 1 indicating no symptom and 5 indicating very severe occurrences of symptom. The second scale (functional disability) consists of five questions (8–12) and is intended primarily to assess walking capacity. Each question is rated on a scale from 1 to 4, with 1 indicating no impairment and 4 indicating greater disability [47,48]. 

#### 2.3.4. Step Activity Monitoring 

Using a pedometer, the distance and the number of steps walked per day were determined. According to USA Centers for Disease Control and Prevention guidelines, the typical average number of steps for most adults is 5000 steps per day, which in most cases is equivalent to about 4 km or 2.5 miles. Many studies have shown that 80 percent of daily steps among less active people have light intensity. Because of this, and to encourage people to increase their amount of moderate to vigorous physical activity, most pedometers set a goal of 10,000 steps per day [49].

#### 2.3.5. Quality of Life Enjoyment and Satisfaction 

The degree of pleasure and satisfaction experienced by patients during the last week in various areas of daily activities was measured using the Quality of Life Enjoyment and Satisfaction Questionnaire (Q-LES-Q-SF). Comprehensive enjoyment and satisfaction with physical health, mood, work, household and leisure activities, social and family relationships, daily functioning, sexual desire, economic status, vision in terms of ability to do work or hobbies, ability to move physically, well-being, treatment, and life contentment were assessed by the patients themselves in 16 items on a 5-point scale. The minimal total score is 16 and the maximal is 80. Total score is expressed as a percentage of the maximum total score across all items (0–100). The lower the score, the less pleasure and satisfaction with life. In many studies, a result of 70% is considered the normal threshold of life enjoyment and satisfaction [50,51].

#### 2.3.6. Laboratory Examination

All patients underwent laboratory examination to assess the level of these indicators: complete blood count with erythrocyte sedimentation rate, rheumatoid factor, IgM, anti-cyclic citrullinated peptide antibodies, C-reactive protein, anti-nuclear antibodies, anti-double-stranded DNA, complements C3 and C4, serum uric acid, serum folate, vitamin B12, 25-hydroxy vitamin D, HbA1, and creatine kinase.

#### 2.3.7. Electroneuromyography Tests

Sensory and motor conduction velocities of the peroneal and tibial nerves were detected bilaterally and F waves in response to tibial nerve stimulation before treatment and at the end of the second month of follow-up.

#### 2.3.8. Magnetic Resonance Imaging Founds

All patients were examined on MRI scanners with a power of 1.5 Tesla or more before surgery, before pharmacotherapy and LTPI treatment, and 4 months after treatment.

### 2.4. Treatment

#### 2.4.1. Pharmacotherapy

All patients underwent a course of therapy using the drugs Etoricoxib 90 mg for 10 days, Tolperisone hydrochloride 150 mg 2 times a day for 15 days, B1, B6, B12 complex vitamins (2.0 mL milgamma) intramuscularly for 5 days, Gabapentin 300 mg 3 times a day for 3 months, and Voltaren gel 2 times a day for 15 days.

#### 2.4.2. Identification of Trigger Points for Injection

The term “trigger point” is often used in the literature to describe myofascial points. In many definitions, it is indicated as pathological changes in muscles, similar to a hypersensitive bundle or nodule of muscle fiber of harder than normal consistency in patients with myofascial pain syndrome [52]. In our work, as a trigger point, we took those points that are very painful and irritable on palpation and that cause reflex spasms in the surrounding muscles, which can be radiated along the spinal column, or the sciatic nerve, towards the zone of reference in addition to the development of recognizable pain (the most acute pain that the patient experiences when moving). We defined trigger points at the levels L4–L5, L5–S1, and in the PSIS projection. For the first time, we identified trigger points in bone tissue. To do this, we palpated the most painful spinous processes and the most painful bony areas in the PSIS projection. We have carefully differentiated “Trigger Points” from “Tender Points”. which are sometimes mistaken for synonyms. In fact, tender points indicate a focal pain directly under the area of palpation, but do not cause referred pain [35].

#### 2.4.3. Intramuscular Lidocaine Trigger Point Injection in L4–S1

**Figure 2:**
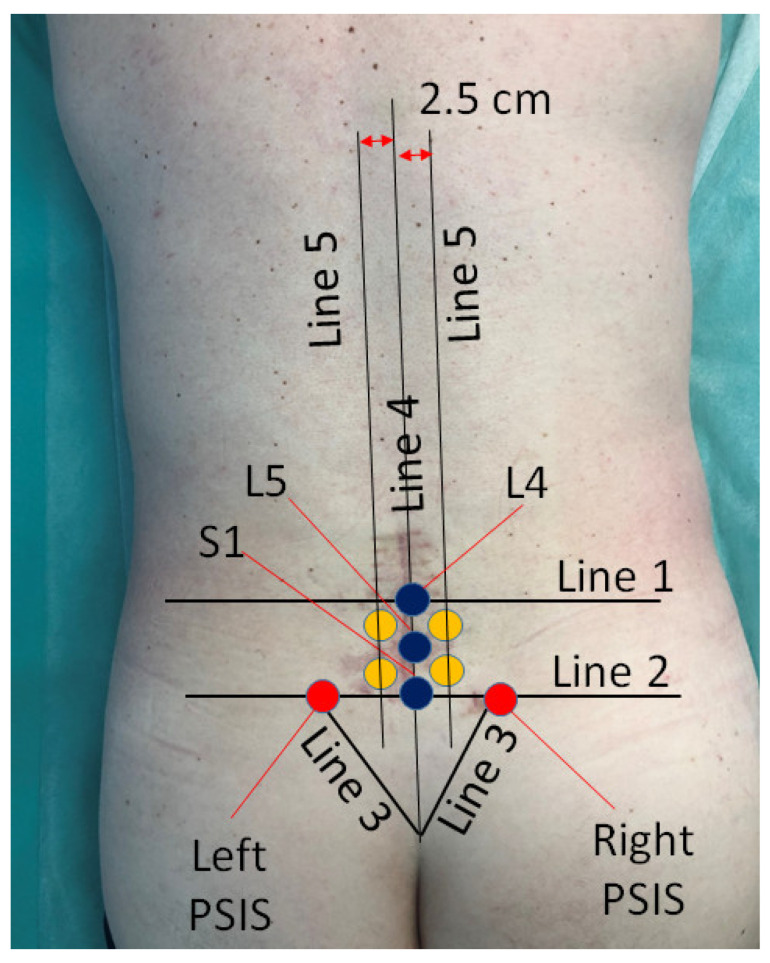
Localization of lidocaine trigger point injection. Notes: PSIS: posterior superior iliac spine; S1: first sacral vertebra; L5: fifth lumbar vertebra; L4: fourth lumbar vertebra; Line 1: Line between iliac crests (intercristal line); Line 2: Line between PSIS; Line 3: edge of sacrum; Line 4: posterior midline; Line 5: paravertebral line; Red points: localization of intramuscular and intraosseous lidocaine trigger point injections in PSIS; Blue points: localization of intraosseous lidocaine trigger point injections in spinous processes L5 and S1; Yellow points: localization of intramuscular lidocaine trigger point injections in L4—L5 and L5–S paravertebral intervals.

Trigger point injection in L4–S1 is a procedure for relieving pain and muscle tension in paravertebral trigger points by injecting 2%–4 mL of lidocaine. The procedure takes about 30 min, is performed on an outpatient basis, and does not require special preparation from the patient. Using palpation, the most painful points are determined at the L4–S1 level, which are most often located within 1–2.5 cm lateral to L4–L5 and L5–S intervals. In this area, an intramuscular needle can be inserted into the multifidus and erector spinae muscles [53]. After treating the skin with an antiseptic solution, the medication is administered, followed by the application of an aseptic dressing. Procedures were carried out 5 times with a time interval between procedures of 3 days (Figure 2).

#### 2.4.4. Intramuscular Lidocaine Trigger Point Injection in PSIS

Trigger point injection in PSIS is a procedure for relieving pain and muscle tension in the PSIS projection by administering 2%–4 mL of lidocaine. The procedure takes about 30 min, is performed on an outpatient basis, and does not require special preparation from the patient. Using palpation, the most painful points above the projection of the PSIS are determined. In this zone, the muscle fibers of the multifidus spinal muscle (musculi multifidi), extending from the medial surface of the PSIS, and the muscle fibers of the gluteus maximus muscle, extending from the lateral-posterior surface of the PSIS are located [54]. It should be noted that according to our ultrasound observations and reports of other authors based on their cadaveric and MRI data, it was established that the origin of the gluteus maximus muscle extends to the medial surface of the PSIS and the sacral spinous processes. Moreover, in these studies, 81 ± 11% of the area between the midline and the PSIS was occupied by the gluteus maximus [55,56]. 

After treating the skin with an antiseptic solution, the medication is administered, followed by the application of an aseptic dressing. Procedures were carried out 5 times with a time interval between procedures of 3 days (Figure 2).

#### 2.4.5. Intraosseous Lidocaine Trigger Point Injection in Spinous Process L5 and S1

Intraosseous LTPI in the spinous process of L5 or S1 is a highly effective analgesic procedure using intraosseous injection of an anesthetic into the spongy substance of the L5 or S1 vertebra. The projection of the spinous process is determined by palpation. After treating the skin with an antiseptic solution, a needle with a mandrel is inserted with a screwing motion perpendicularly into the bone until the spongy substance is reached. Confirmation of the presence of a needle in the spongy substance is bone marrow aspiration. Approximately 2 mL of blood is mixed with medication and injected slowly. The procedure is followed by the application of an aseptic dressing. Procedures were carried out 5 times with a time interval between procedures of 3 days (Figure 2). In difficult cases (obesity, spinal deformity, keloid, and hypertrophic scars), intraosseous ultrasound-guided injection was used.

#### 2.4.6. Intraosseous Lidocaine Trigger Point Injection in PSIS

**Figure 3:**
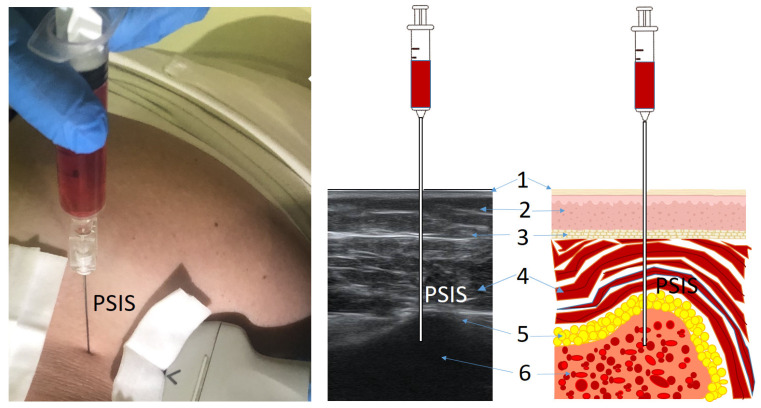
Ultrasound-guided intraosseous lidocaine trigger point injection technique. Notes: PSIS: posterior superior iliac spine; 1: epidermis/dermis; 2: subcutaneous tissue; 3: superior muscle fascia; 4: gluteus maximus; 5: periosteum; 6: spongey substance.

Intraosseous LTPI in PSIS is a highly effective analgesic procedure using intraosseous injection of an anesthetic into the spongy substance of PSIS. The projection of the PSIS is determined by palpation. After treating the skin with an antiseptic solution, a needle with a mandrel is inserted with a screwing motion perpendicularly into the bone until the spongy substance is reached. Confirmation of the position of a needle in the spongy substance is bone marrow aspiration. Approximately 2 mL of blood is mixed with the drug mixture and injected slowly. The procedure is followed by the application of an aseptic dressing. Procedures were carried out 5 times with a time interval between procedures of 3 days. In difficult cases (obesity, spinal deformity, keloid, and hypertrophic scars), intraosseous ultrasound guided injection was used (Figure 2 and Figure 3).

### 2.5. Statistical Analysis

In our study, we used SPSS software for Windows (version 20) to process data analysis. Mean (M), standard deviation (SD), and standard error of the mean (SEM) of the participants’ characteristics were calculated. For normality determination, the Shapiro–Wilk test was used and for testing quality of variances, Levene’s test was used. By multivariate NOVA test, differences between the three groups were tested statistically. The Bonferroni correction test was applied to reduce the chances of obtaining false-positive results (type I errors). An independent group *t* test was used to compare means of the same variable between two groups. The *p* value was set at 0.05.

## 3. Result

### 3.1. Clinical Examination

Before treatment, no symptoms of mental or cognitive disorders were identified in all examined patients. Eye movements were symmetrical within normal limits, and there was no nystagmus. No voice or swallowing disorders were found. There were no motor deficits and no bladder and bowel dysfunction. Pathological reflexes were not elicited. No gait or coordination disturbances were observed. However, other neurological disorders have been identified as pain, paresthesia, bilateral or unilateral sensory deficit in the L5 and/or S root innervation zone, and neurological claudication. There were no side effects after the use of pharmacotherapy and intramuscular and intraosseous LTPI treatment in our study.

### 3.2. Pain Syndrome

Before treatment, pain syndrome on the VAS scale had a high level in all groups and averaged 7.2 ± 0.11 points, with no significant differences between the groups. 

**Figure 4:**
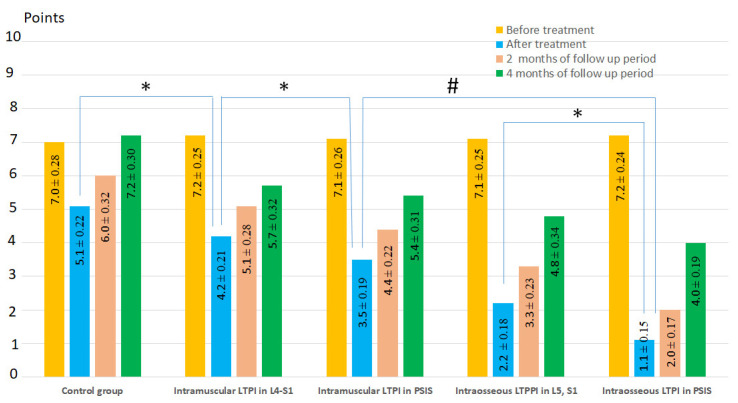
Dynamics of pain syndrome after treatment and in the follow-up period in the control group and lidocaine trigger point injections subgroups. Notes: LTPI: lidocaine trigger point injection; S1: spinous process of the first sacral vertebra; L5: spinous process of the fifth lumbar vertebra; L4–S1: paravertebral intervals between L4–L5 and L5–S1; PSIS: posterior superior iliac spine; # *p* ≤ 0.01; * *p* ≤ 0.05.

After treatment, the reduction in pain was more than two times greater in the LTPI group compared to the control group and averaged 61.5% (t = 12.3, *p* = 0.001). In the long-term period, pain was lower in the LTPI group than in the control group by 38% (t = 5.42, *p* = 0.001) at the end of the second month of follow-up period and by 31% (t = 3.43, *p* = 0.001) at the end of the fourth month of follow-up period (Figure 4). 

**Figure 5:**
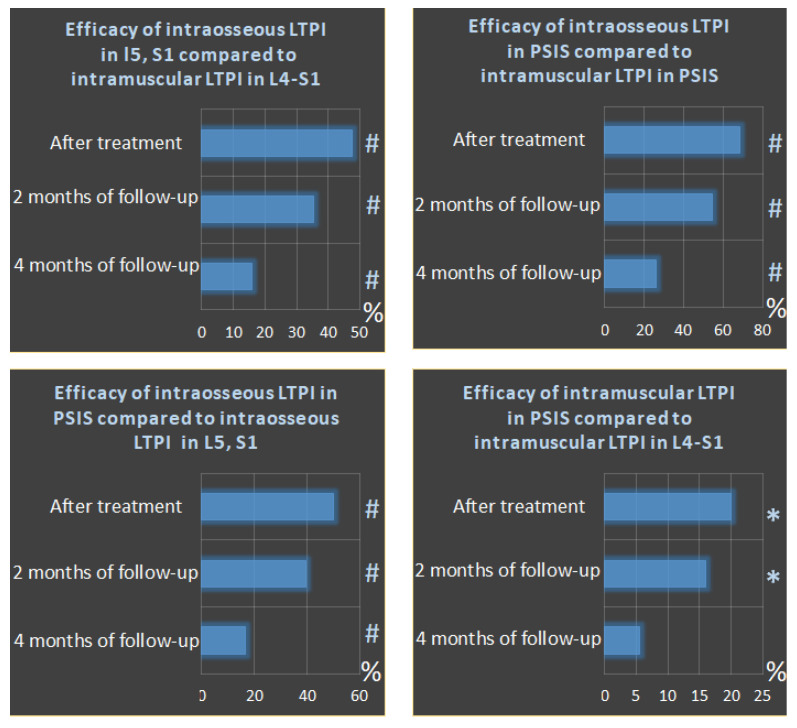
Comparative analysis between LTPI subgroups on the effectiveness of analgesic effects after treatment and in the follow-up period. LTPI: lidocaine trigger point injection; S1: spinous process of the first sacral vertebra; L5: spinous process of the fifth lumbar vertebra; L4–S1: paravertebral intervals between L4–L5 and L5–S1; PSIS: posterior superior iliac spine; #: *p* ≤ 0.01; *: *p* ≤ 0.05.

Intraosseous LTPI had a superior analgesic effect compared with intramuscular LTPI by 57.1% (t = 5.18, *p* = 0.001) after treatment, and this advantage was maintained at second and fourth months of follow-up by 44.2% (t = 4.94, *p* = 0.0001) and 20.7% (t = 2.35, *p* = 0.02), respectively. In addition, it was found that the analgesic effect of intraosseous LTPI in PSIS was higher than in the spinous process of L5 and S1 by 50% (t = 4.69, *p* = 0.0001). It was also found that intramuscular LTPI in PSIS had a greater analgesic effect compared to intramuscular LTPI in the L4–S1 region by 20% (t = 2.47, *p* = 0.02). In the long-term period, the pain symptom at the end of the second and fourth months of follow-up period remains superior after intraosseous LTPI in PSIS compared to intraosseous LTPI in L5 and S1 by 39.4% (t = 4.59, *p* = 0.0001) and 16.7% (t = 2.8, *p* = 0.007), respectively, and after intramuscular LTPI in PSIS compared to intramuscular LTPI in the L4–S1 area by 15.9% (t = 2.47, *p* = 0.02) and 5.6% (t = 1.1, *p* = 0.3), respectively. The analgesic effect of LTPI in the PSIS and spinous process of L5 and S1 was higher when using the intraosseous method compared to the intramuscular method after treatment by 68.6% (t = 8.40, *p* = 0.0001) and 47.6% (t = 7.01, *p* = 0.001), respectively, and at the end of the second month of observation by 54.5 (t = 8.04, *p* = 0.0001) and 35% (t = 6.30, *p* = 0.0001), respectively, and at the end of the fourth month of observation by 25.9% (t = 4.90, *p* = 0.0001) and 15.8% (t = 3.18, *p* = 0.003), respectively (Figure 5). 

### 3.3. Sensory Deficit in L5 and S1 Dermatomes in Foot and Leg

Before treatment, tactile sensations were reduced in all patients compared to unaffected dermatomes L1–L2 on the thigh. A decrease in tactile perception was expressed in the area of innervation of the L5 and/or S1 root. In 76% of cases (*n* = 85), hyposthesia was bilateral. Using a 5-point scale, patients subjectively assessed the level of sensation in the L5 and S1 dermatomes compared to the unaffected L1–L2 dermatomes on the thigh. When two dermatomes L5 and S1 were affected on two limbs, the average value of L5 and S1 hyposthesia on the limb with more pronounced changes was taken into statistical processing. Tactile sensation in the L5 and S1 dermatomes varied from 1 to 4 points and averaged 2.1 ± 0.08 with no significant differences between groups.

**Figure 6:**
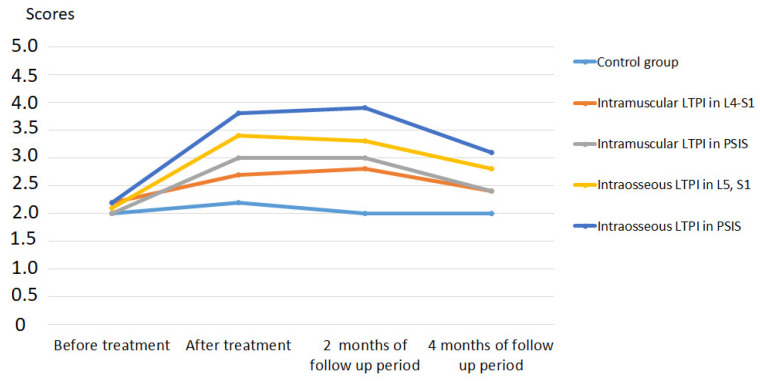
Dynamics of tactile sensation after treatment and in the follow-up period in observation groups. LTPI: lidocaine trigger point injection; S1: spinous process of the first sacral vertebra; L5: spinous process of the fifth lumbar vertebra; L4–S1: paravertebral intervals between L4–L5 and L5–S1; PSIS: posterior superior iliac spine.

**Figure 7:**
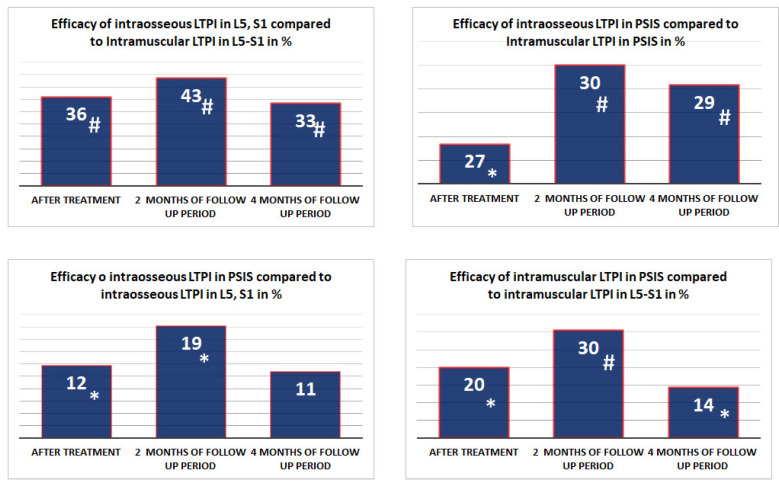
Comparative analysis between LTPI groups on the effectiveness of sensory recovery after treatment and in the follow-up period. LTPI: lidocaine trigger point injection; S1: spinous process of the first sacral vertebra; L5: spinous process of the fifth lumbar vertebra; L4–S1: paravertebral intervals between L4–L5 and L5–S1; PSIS: posterior superior iliac spine; # *p* ≤ 0.01; * *p* ≤ 0.05.

After treatment, a decrease in hypoesthesia was observed in the LTPI group but not in the control group. The recovery of tactile sensation averaged 51.7% (t = 4.80, *p* = 0.001). This result had a prolonged effect with a decreasing trend in the long term. However, the improvement in tactile sensation remained at the level of 52.9% (t = 4.90, *p* = 0.001) at the end of the second month of follow-up period and 25.9% (t = 2.14, *p* = 0.04) at the end of the fourth month of follow-up period (Figure 6). It is noteworthy that the improvement in tactile sensation was more pronounced after the use of intraosseous LTPI compared with intramuscular LTPI after treatment at the end of the second and fourth months of the follow-up period by 26.3% (t = 3.35, *p* = 0.002), 24.1% (t = 2.65, *p* = 0.01), and 22.9% (t = 2.46, *p* = 0.02), respectively. When comparing methods of LTPI treatment with each other after treatment and in the follow-up period, a greater effectiveness of intraosseous LTPI in PSIS was found compared to intraosseous LTPI in the spinous process of S1 by an average of 37.3% (t = 3.20, *p* = 0.003) and intramuscular LTPI in PSIS compared with intramuscular LTPI in L5–S1 on average by 21.3% (t = 2.20, *p* = 0.03) (Figure 7).

### 3.4. Zurich Claudication Questionnaire (ZCQ)

Before treatment, the severity of unpleasant symptoms that develop when walking, such as pain, numbness, weakness, and imbalance (symptom severity), assessed using the ZCO questionnaire, averaged 3.42 ± 0.12 points, and the ability to walk (functional disability)—averaged 2.56. ± 0.11 points. The average values in the control group and LTPI subgroups did not differ significantly from each other (Table 2). 

After treatment, a decrease in the severity of symptoms and functional disability was recorded only in the LTPI group by 30% (t = 3.18, *p* = 0.003) and 38.2% (t = 3.18, *p* = 0.003), respectively, which remained at the level of 29% (t = 2.90, *p* = 0.006) at the end of the second month of follow-up period and 21.4% (t = 2.20, *p* = 0.03) at the end of the fourth month of follow-up period. In the control group after treatment, no significant changes were registered.

**Figure 8:**
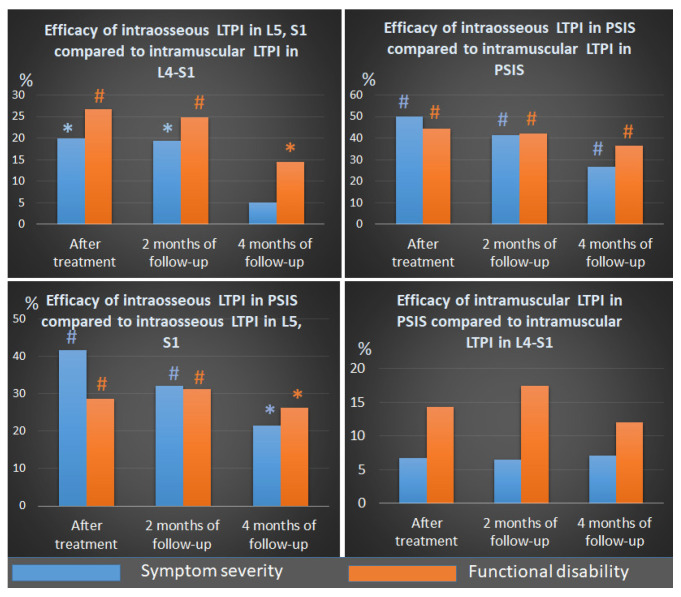
Comparative analysis between LTPI subgroups on the effectiveness of reducing neurogenic classification using the Zurich claudication questionnaire after treatment and in the follow-up period. LTPI: lidocaine trigger point injection; S1: spinous process of the first sacral vertebra; L5: spinous process of the fifth lumbar vertebra; L4–S1: paravertebral intervals between L4–L5 and L5–S1; PSIS: posterior superior iliac spine; # *p* ≤ 0.01; * *p* ≤ 0.05. Blue # and * indicate significance in symptom severity, and orange # and * indicate significance in functional disability.

A comparative analysis of LTPI subgroups shows that intraosseous LTPI can provide a greater reduction in the symptoms severity and functional disability than intramuscular LTPI after treatment by 36.4% (t = 3.53, *p* = 0.001), and at the end of the second and fourth months of the follow-up period by 32.9% (t = 3.18, *p* = 0.003) and 21.8% (t = 2.22, *p* = 0.03) accordingly (Figure 8).

Intraosseous LTPI in PSIS has been shown to be effective in reducing claudication disorders compared with intraosseous LTPI in L5 and S1 by 35.1% (t = 3.71, *p* = 0.0006) after treatment and by 31.6% (t = 2.97, *p* = 0.005) and 23.9% (t = 2.22, *p* = 0.03) at the end of the second and fourth months of follow-up.

The effectiveness of intramuscular LTPI in PSIS and L4–S1 in improving walking did not differ significantly from each other either at post-treatment or during follow-up.

The use of intraosseous LTPI in PSIS was more effective than the use of intramuscular LTPI in PSIS by 47.2% (t = 5.20, *p* = 0.0001) after treatment and by 41.7 (t = 4.45, *p* = 0.0001) and 31.5% (t = 2.97, *p* = 0.005) in the second and fourth months of observation. Also, the use of intraosseous LTPI in L5 and S1 provided greater improvement in walking compared to intramuscular LTPI in the L5–S1 region by 26.7% (t = 2.22, *p* = 0.03) after treatment, which remained at 24.9% (t = 2.06, *p* = 0.045) at the second month of the follow-up period. At the end of the fourth month of follow-up, significant improvements in walking did not differ between subgroups.

### 3.5. Step Activity Monitoring 

**Figure 9:**
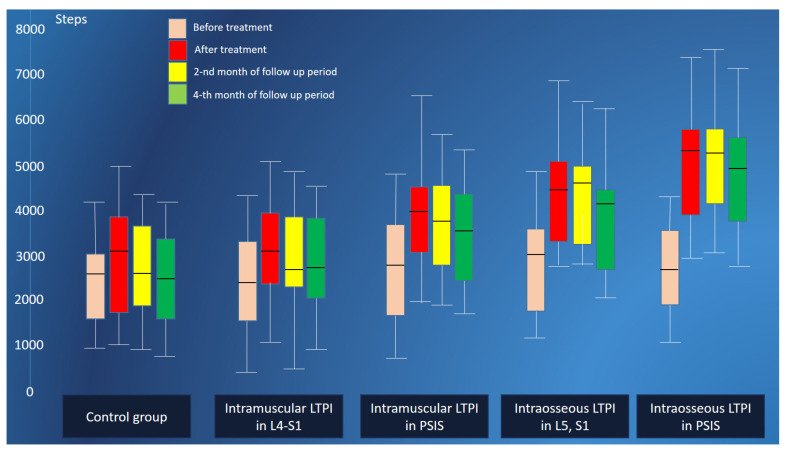
Step activity monitoring of patients in the control group and LTPI subgroups after treatment and in the follow-up period. LTPI: lidocaine trigger point injection; S1: spinous process of the first sacral vertebra; L5: spinous process of the fifth lumbar vertebra; L4–S1: paravertebral intervals between L4–L5 and L5–S1; PSIS: posterior superior iliac spine.

Before treatment, the number of steps per day decreased in the control and LTPI subgroups to 2538 ± 75 steps. The difference between step activity in the control and LTPI subgroups was not significant (Figure 9).

**Figure 10:**
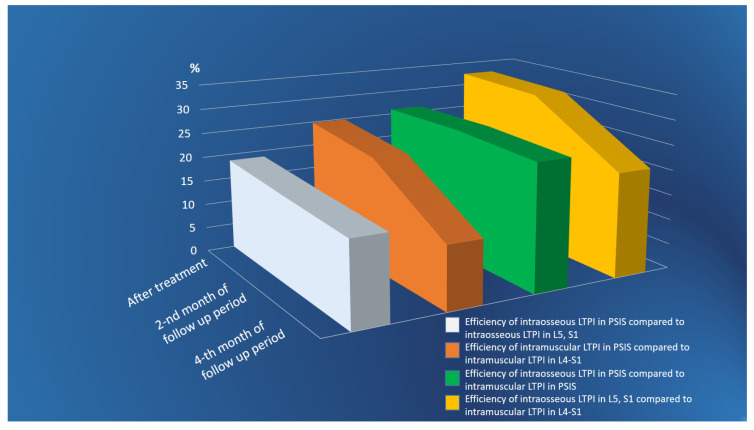
Comparative analysis between LTPI subgroups on the effectiveness of improving daily step activity after treatment and in the follow-up period. LTPI: lidocaine trigger point injection; S1: spinous process of the first sacral vertebra; L5: spinous process of the fifth lumbar vertebra; L4–S1: paravertebral intervals between L4–L5 and L5–S1; PSIS: posterior superior iliac spine.

After treatment, there was an improvement in the step activity of patients after LTPI by 54.3% (t = 10.4, *p* = 0.0001) and after treatment without LTPI by 20.4% (t = 3.50, *p* = 0.001). A more pronounced improvement was noted in patients who underwent a course of intraosseous LTPI, compared with patients after intramuscular LTPI, which amounted to 28.9% (t = 4.70, *p* = 0.0001) after treatment and at the end of the second and fourth months of observation—28.8% (t = 5.96, *p* = 0.0001) and 23.4% (t = 4.37, *p* = 0.0001), respectively. Intraosseous LTPI in PSIS increases step activity after treatment compared to intramuscular LTPI in PSIS by more than 26% (t = 6.50, *p* = 0.0001), and intraosseous LTPI in L5 and S1 compared with intramuscular LTPI in the L5–S1 region by 32% (t = 6.50, *p* = 0.001). Intraosseous LTPI in PSIS was more effective in increasing step activity compared to intraosseous LTPI in L5 and S1 by 18.7% (t = 4.30, *p* = 0.0001) after treatment, with this effect maintained to 17.6% (t = 3.70, *p* = 0.0005) and 16.7% (t = 3.50, *p* = 0.001) at the end of the second and fourth months of follow-up. We also recorded a significant difference in the effectiveness of intramuscular LTPI in PSIS compared with intramuscular LTPI in the L5–S1 region, which was 24.9% (t = 4.20, *p* = 0.0001) after treatment and 22.7% (t = 3.79, *p* = 0.0005) and 12.7% (t = 2.10, *p* = 0.04) at the end of the second and fourth months of the follow-up (Figure 10).

### 3.6. Quality of Life Enjoyment and Satisfaction 

**Figure 11:**
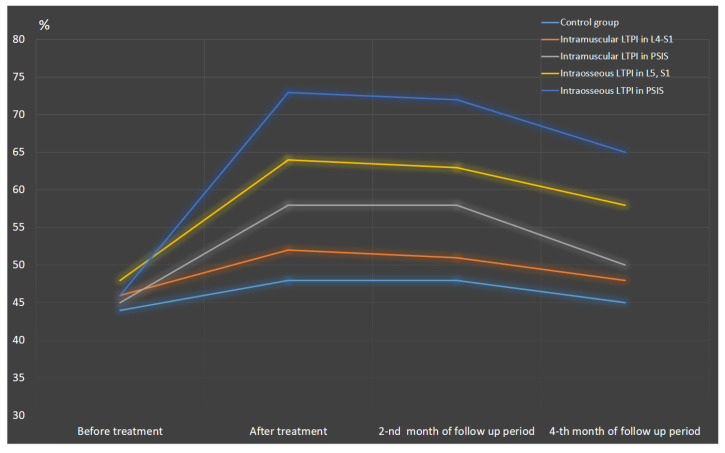
Dynamics of quality of life, determined using the quality of life enjoyment and satisfaction scale after treatment and during the follow-up period in all observed groups. LTPI: lidocaine trigger point injection; S1: spinous process of the first sacral vertebra; L5: spinous process of the fifth lumbar vertebra; L4–S1: paravertebral intervals between L4–L5 and L5–S1; PSIS: posterior superior iliac spine.

The degree of pleasure and satisfaction assessed before treatment using the Q-LES-Q-SF scale was low in all patients and averaged 45.8 ± 0.56%. No significant differences were recorded between groups (Figure 11).

After treatment, the quality of life in LTPI subgroups was higher than in the control group by 28.6% (t = 8.57, *p* = 0.0001), maintaining this improvement at 27% (t = 7.20, *p* = 0.0001) and 22.8% (t = 4.45, *p* = 0.0001) until the end of the second and fourth months of the follow-up period. In accordance with the results obtained above, intraosseous LTPI increased the level of quality of life by 24.5% higher compared to intramuscular LTPI. Accordingly, intraosseous LTPI in PSIS was more effective in improving the quality of life compared to intramuscular LTPI in PSIS by 25.8% (t = 3.81, *p* = 0.0005) and intraosseous LTPI in L5 and S1 compared with intramuscular LTPI in the L4–S1 region by 23.1% (t = 3.39, *p* = 0.002). It is noteworthy that the improvement in quality of life had a prolonged effect, lasting at least four months, exceeding the pre-treatment baseline level by 25.5% (t = 3.82, *p* = 0.0005).

**Figure 12:**
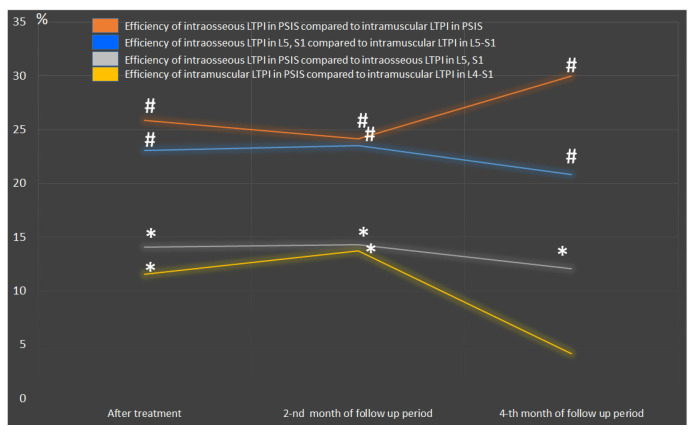
Comparative analysis between LTPI subgroups on the effectiveness of improving quality of life after treatment and in the follow-up period. LTPI: lidocaine trigger point injection; S1: spinous process of the first sacral vertebra; L5: spinous process of the fifth lumbar vertebra; L4–S1: paravertebral intervals between L4–L5 and L5–S1; PSIS: posterior superior iliac spine; #: *p* ≤ 0.01; *: *p* ≤ 0.05.

It was found that the quality of life after intraosseous LTPI in PSIS was superior to LTPI in the L5 and S1 by 14.1% (t = 2.54, *p* = 0.015), which remained at 14% (t = 2.26, *p* = 0.03) at the end of follow-up. No significant differences were found between intramuscular LTPI in the PSIS and the L4–S1 region after treatment (t = 1.67, *p* = 0.09). However, at the end of second-month follow-up, the quality of life of patients who underwent intramuscular LTPI in PSIS was 14% (t = 2.06, *p* = 0.045) higher (Figure 12).

### 3.7. Electroneuromyography Findings

Our study did not include patients whose ENMG revealed signs of severe distal polyneuropathy or damage to the peroneal nerve in the peroneal canal or the tibial nerve in the tarsal canal. In 43.4% (*n* = 49) of patients, the amplitude of compound muscle action potential (CMAP) of the peroneal nerve at the active point of extensor digitorum brevis muscle decreased by an average of 41%. In most cases (78%, *n* = 38), these changes were unilateral, and in 92% (*n* = 45), they were detected on the side of maximum pain irradiation. A decrease in the CMAP amplitude at the active point of the tibialis anterior muscle in response to peroneal nerve stimulation was registered in only 5 patients.

Other electroneuromyography abnormalities have been found by examining the F-wave response to tibial nerve stimulation. These changes were recorded in 94% (*n* = 105) of patients and consisted of a loss and decrease in the amplitudes of responses, an increase in latency of responses with prolongation of F-wave duration, and the registration of repeated responses (temporal dispersion). In 89% (*n* = 93) of cases, F-wave abnormalities were recorded bilaterally with a large deviation from the norm on the side of maximum irradiation of pain. In 95% of cases (*n* = 100), F waves were recorded accompanied by A waves.

After treatment, the characteristics and values of ENMG improved only in 14% (*n* = 6) of patients who underwent a course of intramuscular LTPI and in 27% (*n* = 12) of patients after intraosseous LTPI. However, these changes are moderate in nature and have various aspects, such as increased CMAP amplitude, increased F-response amplitude, reduced temporal dispersion of F-wave, and decreased multiple A-waves, which cannot be compared collectively and are not reliable if compared separately.

### 3.8. Magnetic Resonance Imaging Examination

All patients included in this study had central lumbar spinal stenosis, predominantly at the L4–S1 level, as this was specified in the inclusion criteria. Characteristic features of central lumbar canal stenosis are observed on sagittal images, such as a smoothly defined waist or hourglass shape, and on axial images, such as a trefoil or circumferentially narrowed nerve canal. The severity of spinal stenosis was absence or mild, with a narrowing of the spinal canal and no more than 1/3 of the space available for neural elements [57]. In almost all patients, MRI revealed various bone abnormalities associated with lumbar canal stenosis, such as spondylolysis, spondylolisthesis, end plate irregularities, sclerosis, facet joint osteoarthritis, articular process hypertrophy, vacuum phenomenon of discs and joints, as well as subchondral and synovial cysts.

**Figure 13:**
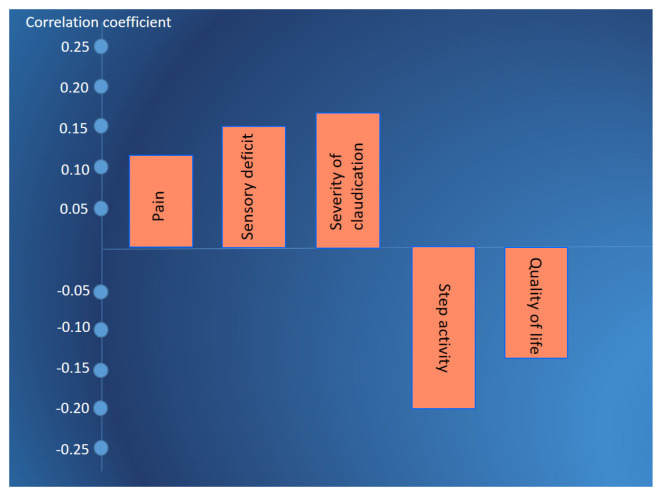
Coefficient correlation between the severity of spinal stenosis, determined by MRI and pain on the visual analogue scale, sensory deficit of L4–S1 dermatomes on a 5-point scale, severity of claudication by the Zurich claudication questionnaire, step activity, and the quality of life by quality of life enjoyment and satisfaction scale in the examined patients up to pharmacotherapy and LTPI treatment. Notes: LTPI: lidocaine trigger point injection; S1: spinous process of the first sacral vertebra; L5: spinous process of the fifth lumbar vertebra; L4–S1: paravertebral intervals between L4–L5 and L5–S1; PSIS: posterior superior iliac spine.

Correlation analysis between the severity of spinal canal stenosis, determined using MRI, and the severity of pain, sensory deficit, step activity, and quality of life revealed a weak correlation between morphological and clinical changes in the examined patients (Figure 13).

**Figure 14:**
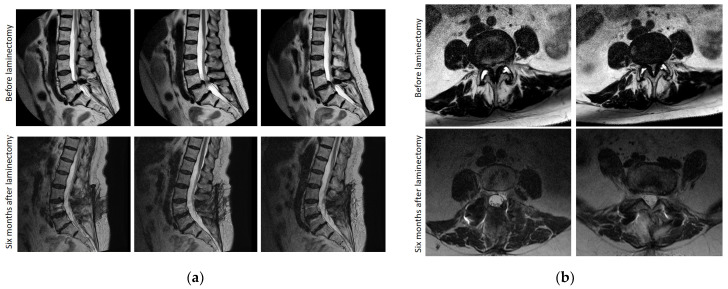
Sequential sagittal T2-weighted (**a**) and sequential axial T2-weighted (**b**) MRI of the lumbosacral spine with the development of severe spinal canal stenosis at the L4–L5 level, exceeding 90% before laminectomy, with complete resolution of the stenosis after surgery treatment in a 59-year-old woman. Despite good postoperative results, pain persists up to 9 points on VAS with a lack of tactile sensation up to 2.5 points in the L5, S1 dermatomes on the left leg and up to 3.5 points on the right leg. At the same time, the step activity averaged 2300 steps per day; symptom severity and functional disability of claudication according to the Zurich claudication questionnaire were 2.9 and 2.1 points, respectively, with a marked decrease in quality of life of up to 61%.

**Figure 15:**
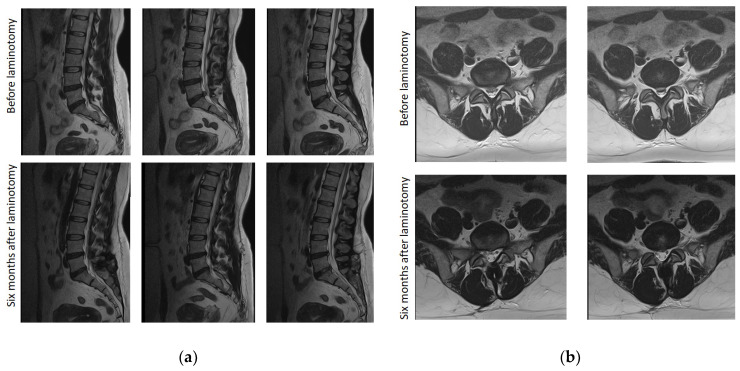
Sequential sagittal T2-weighted (**a**) and sequential axial T2-weighted (**b**) MRI of the lumbosacral spine with the development of severe spinal canal stenosis at the L5–S1 level, exceeding 60% before laminotomy, with complete resolution of the stenosis after surgery treatment in a 55-year-old woman. Despite good postoperative results, pain persists up to 9 points on VAS with a lack of tactile sensation up to 3 points in the L5, S1 dermatomes on the left leg and up to 3.5 points on the right leg. At the same time, the step activity averaged 2950 steps per day, symptom severity and functional disability of claudication according to the Zurich claudication questionnaire were 2.0 and 1.8 points, respectively, with a marked decrease in quality of life of up to 69%.

In many cases, highly effective postoperative decompression of the spinal canal was not accompanied by a sufficient improvement in the clinical status of patients, regardless of the type of surgical intervention (Figure 14 and Figure 15).

## 4. Discussion

Our study is the first to examine the effectiveness of LTPI in the treatment of residual neurological disorders after degenerative lumbar spinal stenosis decompression surgery at the L4–S1 level. In all included patients, the severity of spinal canal stenosis after decompression surgery decreased by less than 1/3 of the normal size of the spinal canal, and in 53.3% (*n* = 65) of cases the lumen of the canal was completely restored. Despite successful decompression of spinal stenosis, many patients continue to experience severe pain, sensory disturbances, and claudication, which negatively affects their daily step activity and quality of life more than 6 months after surgery.

All patients suffered from residual pain after DLSS decompression surgery for more than 6 months but less than 3 years. The localization of the maximum pain syndrome is in the lumbosacral joint. Before treatment, the severity of residual pain on the visual analogue scale (VAS) exceeded 6 points and averaged 7.3 ± 0.11 points. 

In all patients, palpation of the muscles of the back and gluteal region revealed painful points resembling trigger points. These points were most localized in the L4–S1 region, the gluteal region, and in the projection of the PSIS bilaterally, and were more pronounced in the area of pain irradiation. The maximum painful point is determined in the projection of the PSIS on the side of pain irradiation.

In our research, we studied the reflexogenic effect of lidocaine on various trigger points in the muscles and bones of the lumbosacral region. A comparative analysis of intramuscular and intraosseous administration of LTPI in combination with pharmacotherapy compared with the use of pharmacotherapy alone was carried out. In particular, intramuscular injections were performed into the paraspinal muscles at the level of L4–S1 and in the gluteus maximus in PSIS projection (in some cases, injections were carried out into the tendons or periosteum), and intraosseous LTPI—into the spongy substance of L5 or S1 (in patients after laminotomy) and PSIS.

### 4.1. High Efficiency of LTPI 

Our results proved that the use of LTPI enhances the analgesic effect of pharmacotherapy by 1.3 times and only leads to a significant recovery of sensory functions, regression of neurogenic claudication, and improvement in quality of life. This advantage remained at the same level for 2 months, with a gradual decrease to 55% by the end of the fourth month of observation.

#### 4.1.1. Analgesic Effect of LTPI

Currently, the existing literature exhibits contradictory findings concerning the effectiveness of LTPI compared to pharmacotherapy. Nevertheless, results similar to ours were obtained by other authors as a result of studying the effectiveness of intramuscular and intraosseous LTPI in the treatment of patients with low back pain associated with lumbar disc herniation [26,58,59], as well as in the treatment of patients with DLSS [60,61]. According to A.O. Kosak, LTPI was superior to intravenous NSAIDs in the treatment of acute low back pain by 57.7% [62].

Other studies have examined the effectiveness of dry needling [63,64], steroid injections [61], steroid-lidocaine injection [65], normal saline injection [66], and sterile water injections [67]. All works noted the high efficiency of these methods. However, a comparative analysis between them and LTPI has not been carried out, with the exception of one study, which showed a greater analgesic effect of LTPI compared with sterile water needling for 1 month after injection [67]. It is important to note that in many studies, the addition of glucocorticoids to LTPI did not increase the analgesic effect of LTPI [68]. 

The high analgesic effect of LTPI is associated with multifactorial mechanisms of inhibition of pain modulation at the segmental and suprasegmental levels, as well as with local action [25,69]. 

The primary mechanism of lidocaine local anesthesia is the blocking of voltage-gated Na+ channels (VGSC/NaV), which reduces Na+ channel peak currents and accelerates the deactivation process, reducing neuronal excitability and thereby preventing or reducing the sensation of pain [27]. Moreover, lidocaine can block K+ ion channels and regulate intracellular and extracellular calcium concentrations through other ligand-gated ion channels [27]. Additionally, the local anesthetic effect of lidocaine is associated with the decrease of pain neurotransmitter concentrations as calcitonin gene-related peptide [70], substance P [71,72], and bradykinin [73], which normalize the threshold of excitability of nociceptive receptors. It is important to note that the anti-inflammatory properties of lidocaine are crucial for reducing trigger point activity by inhibiting the secretion of tumor necrosis factor-alpha (TNF-α) in human leukocytes activated by lipopolysaccharide [74].

The main segmental mechanism of LTPI, according to the gate control theory of pain [23,31], is a decrease in the afferentation of unmyelinated sensory fibers that inhibit substantia gelatinosa of Rolando, which plays the role of an inhibitory gate for nociceptive afferentation into the dorsal horn of the spinal cord. Thus, due to the inhibition of peripheral nociception and the activity of dorsal horn neurons, the excitability of the central nervous system decreases [75,76].

Small-volume Injections of fluid can stimulate acupuncture points that coincide with or are close to active trigger points. As a result, LTPI can enhance the secretion of endogenous opioids and β-endorphin in the brain, producing a potent descending analgesic effect [39] and increasing the accumulation of enkephalin, which inhibits the transmission of nociceptive afferentation in the central nervous system [77].

In our study, the analgesic effect of LTPI lasted without negative dynamics for 2 months and decreased by half at the end of the fourth month of follow-up. In other works, the prolonged analgesic effect of LTPI lasted for 3 months [78,79]. The prolonged analgesic effect of LTPI is associated primarily with a decrease in both peripheral and central sensitization due to temporary inhibition of trigger point activity and a decrease in the intensity and duration of peripheral nociceptive afferentation [23,31]. However, the analgesic effect has a short-term effect due to the persistence of morphological abnormalities in the spine, due to which afferentation of nociceptive impulses continues until peripheral and central sensitization is reformed [29].

In clinical practice, a close connection has been established between the activity of trigger points and hypertonicity of the lower back and gluteal muscles. Hence, reducing the tension of the parasinalis and periform muscles by LTPI leads to a decrease in compression of the paravertebral and sciatic nerves. In addition, reflexogenic normalization of muscle tone leads to decompression of the intervertebral discs and thereby to a decrease in protrusion into the spinal canal. Other studies have found that reflexogenic segmental improvement of blood circulation enhances and accelerates the processes of restoration of damaged nerves and muscles due to the accumulation of growth factors. Moreover, one of the reasons for the development of residual neurological disorders after DLSS is postoperative lumboparaspinal compartment syndrome. The use of LTPI in the area of damaged muscles can significantly reduce the pathogenetic cause of pain and functional disability in these patients [26,36].

#### 4.1.2. Recovery of Sensory Function

Improvement in sensory function after a course of LTPI occurs mainly due to improved sciatic nerve conduction as a result of reduced muscle tension in the piriformis muscle [80]. In addition, a decrease in muscle tone of the paravertebral muscles and an increase in the distance between the vertebrae free the L5 and S1 roots from pinching and promote their recovery [81]. Many patients, after undergoing LTPI and pain reduction, immediately experience a noticeable improvement in tactile sensations in the area of pain irradiation [6,82,83,84]. A number of authors believe that patients with severe pain develop hyposensitivity of the somatosensory system to nonnoxious mechanical stimuli due to central sensitization [85,86,87,88]. This functional hypoesthesia is reversible and regresses after the pain subsides [82,89]. 

#### 4.1.3. Regression of Neurogenic Claudication and Improvement of Daily Step Activity

Although DLSS decompression surgery is highly effective in reducing pain and symptoms of neurogenic claudication in most patients [90,91], up to 40% of patients report persistent walking disability following DLSS decompression surgery [92]. However, in our study, under inclusion conditions, all patients suffered from symptoms of neurogenic claudication. An average 34% reduction in neurogenic claudication after completion of an LTPI course and a 54.9% improvement in daily walking ability are associated with several interrelated reflexogenic mechanisms of the LTPI. In addition to reducing pain, LTPI improves microcirculation at the injection site and in the muscles located in the segmental zone of innervation. This effect can be achieved directly through reflex vasodilation or indirectly through relaxation of tense muscles compressing small vessels in the affected area [93]. Furthermore, relaxation of gluteal and lumbar muscles in patients with chronic pain has a positive effect on the endurance of the muscles involved in walking and on postural reflexes [81]. No studies were found in the literature on the effectiveness of LTPI in improving gait and reducing neurogenic claudication. However, a 45-year-old paper was found demonstrating that the analgesic effect and interruption of the pain cycle of LTPI can improve exercise capacity of the leg without improving circulation in patients with intermittent claudication [94].

Reduction in neurogenic claudication and improvement of daily step activity after LTPI treatment was short-term and continued without significant dynamics during the first 2 months. However, at the end of the fourth month, the severity of neurogenic claudication was only 21.4% below baseline, and daily step activity was 37.4% higher than pre-treatment levels. In our opinion, the main reason for the long-term neurogenic claudication regression and daily step activity improvement is the reflexogenic action of LTPI, manifested in a decrease in muscle hypertonicity, improvement of local and regional microcirculation, increase in blood flow in the vessels of the lower extremities, normalization of postural reflexes, and increase in muscle endurance to hypoxia in the majority of patients with residual neurological deficit after DLSS [95]. Correlation analysis revealed a strong correlation between the severity of pain and the degree of neurogenic claudication (r = 0.55, *p* < 0.05). This is explained by the fact that the majority of patients with neurogenic claudication prioritized walking a short distance with reduced pain over walking farther with pain. It is important to note the negative impact of comorbidity, smoking status, and complications on pain intensity, functional recovery, and daily step activity after DLSS decompression surgery [96].

### 4.2. Features of the Use of Intraosseous LTPI

In this study, we first examined the effectiveness of intraosseous LTPI compared with traditional intramuscular LTPI in the treatment of patients with residual neurological disorders after DLSS decompression surgery. The analgesic effect of intraosseous LTPI was superior to intramuscular by 66.4% (*p* < 0.01) immediately after treatment and by 87.1% (*p* < 0.01) and 71.6% (*p* < 0.01) at the end of the second and fourth months of follow-up. The high efficiency of intraosseous LTPI is associated with the features of the receptor apparatus in the injection zone. The concentration of slow fibers in the spongy substance is much higher than in muscle trigger points. Moreover, in spongy substance the ratio of slow unmyelinated C-fibers to fast myelinated A-fibers is two to one [97]. In addition, the distribution of lidocaine when administered into the spongy substances is much greater and faster than when administered intramuscularly. According to the “Osteogenic theory of neuro-orthopedic diseases”, the development of pain syndrome in osteochondrosis and the formation of active trigger points in the projections of bone structures such as the spinous processes and PSIS are based on the difficulty of venous outflow from bone as a result of focal disruption of endochondral ossification [98,99]. Thus, increased intraosseous pressure can be considered the cornerstone of the development of pain in patients with osteochondrosis [44,100]. It has been established that an increase in intraosseous pressure entails stimulation of some intraosseous receptors and a decrease in the threshold of excitability of others, while massive and prolonged excitation can lead to the formation of a focus of intraosseous peripheral sensitization [23]. However, given that most afferents consist of slow fibers, stimulation of intraosseous receptors deeply inhibits the cells of gelatinous substances, which cannot then segmentally interfere with nociceptive afferentation [23,31].

It is important to note that the intraosseous LTPI technique contains a therapeutic effect even without lidocaine infiltration due to trepanation of bone tissue, leading to a decrease in intraosseous pressure. Moreover, the participation of reflexogenic and acupuncture mechanisms is not excluded [23,101]. Accordingly, a more pronounced analgesic effect of intraosseous LTPI was accompanied by a more pronounced recovery effect. Although the efficiency of intraosseous LTPI was higher than intramuscular LTPI in regression of sensory deficit and neurogenic claudication and in improving daily step activity immediately after treatment by 88% (*p* < 0.001), 150% (*p* < 0.001), and 68% (*p* < 0.001) sincerely, at the end of the second month of follow-up by 77% (*p* < 0.01), 66.3% (*p* < 0.01), and 179% (*p* < 0.001) sincerely, and at the end of the fourth month of follow-up by 160% (*p* < 0.001), 183% (*p* < 0.001), and 67% (*p* < 0.01) sincerely

### 4.3. Comparative Analysis of LTPI in PSIS and in the L4–S1 Region

A very important result of our work is a comparative analysis of LTPI in PSIS and LTPI in the L4–S1 region. It was found that the analgesic and recovery effects of intramuscular LTPI in PSIS were greater than in the L4–S1 region by 21.7% (*p* < 0.01) and 35% (*p* < 0.01) immediately after treatment and by 30.4% and 44% at the end of the second month of follow-up and by 15% (*p* < 0.05) and 24% (*p* < 0.01) at the end of the fourth month of follow-up. Approximately the same results were obtained when studying intraosseous LTPI in PSIS and the spinous processes of L5 and S1. Intraosseous LTPI turned to be more effective in reducing pain and recovery of neurological disorders by 22.7 (*p* < 0.01) and 35% (*p* < 0.01) immediately after treatment, by 34.9 and 27.9% at the end of the second month of follow-up, and by 17% (*p* < 0.05) and 41% (*p* < 0.01) at the end of fourth months of follow up. According to the analysis of the results obtained, LTPI carried out in the PSIS projection has a more pronounced therapeutic effect than LTPI provided in the L4–S1 region. Especially when using intramuscular LTPI due to the large difference between the ratio of unmyelinated to myelinated sensory fibers in PSIS and the paraspinal trigger point, which is higher in PSIS. This large difference is most likely due to the relatively high concentration of unmyelinated fibers in the PSIS projection due to the presence of muscle tendons and periosteum at the injection site in addition to muscle fibers [6]. It is difficult to confirm the presence of a difference in the ratio of unmyelinated and myelinated sensory fibers in the spongy substance of the PSIS and the spinous process. However, there are large differences in the size of the bony structures between the PSIS and the spinous process. Thus, with a large difference in the area of action of lidocaine on intraosseous receptors and afferents in PSIS compared to the area of its influence on the spinous process, the analgesic effect of LTPI in PSIS is undoubtedly higher. 

### 4.4. Efficacy of Lidocaine Compared with Other Local Anesthetics in Trigger Point Inactivation

In our study, we did not compare the effectiveness of lidocaine with other anesthetics in the treatment of patients with residual pain after DLSS decompression surgery, since this was not our objective. In addition, such a comparison of different anesthetics in the treatment of patients on this topic has not previously been made. However, there are many comparative clinical studies in the literature of lidocaine and other anesthetics in the treatment of other pain syndromes using trigger point injections.

Comparative analysis of lidocaine and mepivacaine in the treatment of myofascial pain found that both local anesthetics were equally effective in treating myofascial pain by trigger point injection. However, the mepivacaine-treated group exhibited significantly lower post-injection tenderness than the lidocaine group [102]. Other studies have found no significant difference between LTPI and bupivacaine trigger injection in the treatment of head and neck myofascial pain syndrome [25,103]. It was not possible to identify scientific publications on the comparative analysis of lidocaine with levobupivacaine and ropivacaine. However, in other studies, comparative statistical analysis of trigger point injections of levobupivacaine and ropivacaine did not reveal significant differences between groups in pain during injection, treatment effectiveness, or duration of pain relief in myofascial pain syndrome management [104]. Thus, according to the majority of authors, there is an unreliable difference between various anesthetics in the treatment of pain using trigger point injections. Many authors suggest that relief of pain is mainly due to reflex mechanisms rather than to the pharmacological effects of the injected solutions [105].

## 5. Conclusions

LTPI has been proven to be highly effective in the treatment of patients with residual neurological disorders after DLSS decompression surgery, which is manifested not only by a high analgesic effect but also by a clear regression of sensory deficits with improved gait, daily step activity, and quality of life, not only during the course of treatment but also continues for at least 2 months after treatment. Intraosseous LTPI is more effective than intramuscular LTPI by 92%. For the first time, LTPI in PSIS was found to be 28.6% more effective than LTPI in L4–S1. Considering the safety, simplicity, low cost, minimal side effects, multifactorial mechanisms of pathogenesis, and prolonged effect, it is recommended to use this method in the treatment of patients with residual neurological pain after DLSS decompression surgery and various forms of low back pain.

## 6. Declaration of Patient Consent

The authors confirm that they have obtained all necessary patient consent forms. In the form, the patients gave their consent for the publication of their images and other clinical information in the journal. The patients understand that their names and initials will not be published, and appropriate steps will be taken to conceal their identity, but anonymity cannot be guaranteed.

## Figures and Tables

**Table 1:** Demographic and clinical characteristics of the participants.

	Control Group	LTPI Group				
		Intramuscular LTPI PSIS Subgroup	Intramuscular LTPI L4–S1 Subgroup	Intraosseous LTPI PSIS Subgroup	Intraosseous LTPI In L5, S1 Subgroup	*p*
No	21	20	21	21	20	
Age (years)	53.0 ± 5.12	53.3 ± 4.80	54.1 ± 4.90	53.2 ± 4.98	53.7 ± 5.18	*p* > 0.05
Gender (female:male)	10:10	10:9	10:10	10:10	10:10	*p* > 0.05
Disease duration after DLSS decompression surgery (months)	22.4 ± 8.20	20.2 ± 7.37	21.4 ± 7.70	21.9 ± 7.84	22.0 ± 8.08	*p* > 0.05
Pain by 10-point VAS (points)	7.86 ± 1.15	7.78 ± 1.22	7.95 ± 1.18	7.69 ± 1.20	7.80 ± 1.25	*p* > 0.05

Note: LTPI— lidocaine trigger point injection; PSIS—posterior superior iliac spine; L5–S1—parvertebral space between the fifth lumbar and first sacral vertebrae; S1—first sacral vertebra; L5—fifth lumbar vertebra; DLSS—degenerative lumbar spinal stenosis; VAS—visual analogue scale; *p*—level of marginal significance, Mean ± SEM.

**Table 2:** Assessment of neurogenic claudication using the Zurich Claudication Questionnaire before and after treatment in the observed groups.

	Before Treatment	After Treatment	2 Months of Follow-up Period	4 Months of Follow-up Period
	Symptom severity			
Control group	3.4 ± 0.18	3.3 ± 0.18	3.3 ± 0.19	3.4 ± 0.19
Intramuscular LTPI in L4–S1	3.4 ± 0.19	3.0 ± 0.22	3.1 ± 0.21	3.3 ± 0.18
Intramuscular LTPI in PSIS	3.5 ± 0.17	2.8 ± 0.18	2.9 ± 0.18	3.0 ± 0.19
Intraosseous LTPI in L5, S1	3.4 ± 0.19	2.4 ± 0.21	2.5 ± 0.19	2.8 ± 0.20
Intraosseous LTPI in PSIS	3.4 ± 0.19	1.4 ± 0.17	1.7 ± 0.19	2.2 ± 0.19
	Functional disability			
Control group	2.6 ± 0.17	2.5 ± 0.19	2.7 ± 0.19	2.7 ± 0.17
Intramuscular LTPI in L4–S1	2.5 ± 0.16	2.1 ± 0.17	2.3 ± 0.17	2.5 ± 0.16
Intramuscular LTPI in PSIS	2.6 ± 0.18	1.8 ± 0.17	1.9 ± 0.17	2.2 ± 0.18
Intraosseous LTPI in L5, S1	2.6 ± 0.16	1.4 ± 0.14	1.6 ± 0.14	1.9 ± 0.15
Intraosseous LTPI in PSIS	2.5 ± 0.17	1.0 ± 0.15	1.1 ± 0.15	1.4 ± 0.16

Note: LTPI: lidocaine trigger point injection; S1: spinous process of the first sacral vertebra; L5: spinous process of the fifth lumbar vertebra; L4–S1: paravertebral intervals between L4–L5 and L5–S1; PSIS: posterior superior iliac spine.

## Data Availability

Not available.

## References

[1] Sachs B., Fraenkel J. (1900). Progressive ankylotic rigidity of the spine (spondylose rhizomelique). J. Nerv. Ment. Dis..

[2] Deer T., Sayed D., Michels J., Josephson Y., Li S., Calodney A.K. (2019). A Review of Lumbar Spinal Stenosis with Intermittent Neurogenic Claudication: Disease and Diagnosis. Pain Med..

[3] Trefilova V.V., Shnayder N.A., Novitsky M.A., Ovdienko O.A., Nurgaiev Z.A. (2023). Application pf patient reported outcomes in back pain in adults: Part 2. Pers. Psychiatry Neurol..

[4] Yabuki S., Fukumori N., Takegami M., Onishi Y., Otani K., Sekiguchi M., Wakita T., Kikuchi S., Fukuhara S., Konno S. (2013). Prevalence of lumbar spinal stenosis, using the diagnostic support tool, and correlated factors in Japan: A population-based study. J. Orthop. Sci..

[5] Al-Zami M.K. (2004). Clinical and Morphological Correlations in Neurological Manifestations of Lumbar Osteochondrosis. Ph.D. Dissertation.

[6] Mansur N.Y., Al-Zamil M.K. (2021). Clinical and morphological correlation in the study of 117 patients with lumbar severe pain. Clin. Neurol..

[7] Jackson R.P., McManus A.C., Moore J. (2012). Lumbar spinal stenosis: Treatment options for an aging population. Mo. Med..

[8] Fraser J.F., Huang R.C., Girardi F.P., Cammisa F.P. (2003). Pathogenesis, presentation, and treatment of lumbar spinal stenosis associated with coronal or sagittal spinal deformities. Neurosurg. Focus.

[9] Kalichman L., Cole R., Kim D.H., Li L., Suri P., Guermazi A., Hunter D.J. (2009). Spinal stenosis prevalence and association with symptoms: The Framingham Study. Spine J..

[10] Ferretti F., Coluccia A., Gusinu R., Gualtieri G., Muzii V.F., Pozza A. (2019). Quality of life and objective functional impairment in lumbar spinal stenosis: A protocol for a systematic review and meta-analysis of moderators. BMJ Open.

[11] Fessler R.G. (2021). Surgery versus nonsurgery for lumbar spinal stenosis: An in-depth analysis of the 2016 Cochrane analysis, the studies included for analysis, and Cochrane methodology. J. Neurosurg. Spine.

[12] Pietrantonio A., Trungu S., Famà I., Forcato S., Miscusi M., Raco A. (2019). Long-term clinical outcomes after bilateral laminotomy or total laminectomy for lumbar spinal stenosis: A single-institution experience. Neurosurg. Focus.

[13] Grasso G., Landi A. (2017). Preliminary experience with lumbar facet distraction and fixation as treatment for lumbar spinal stenosis. J. Craniovertebr. Junction Spine.

[14] Hara N., Oka H., Yamazaki T., Takeshita K., Murakami M., Hoshi K., Terayama S., Seichi A., Nakamura K., Kawaguchi H. (2010). Predictors of residual symptoms in lower extremities after decompression surgery on lumbar spinal stenosis. Eur. Spine J..

[15] Gray C.M., Kumar S. (2020). Complete resolution of chronic pain, sensory impairment, and motor dysfunction following percutaneous transforaminal endoscopic decompression in a failed back surgery syndrome patient-a case report. J. Spine Surg..

[16] Martens F., Vajkoczy P., Jadik S., Hegewald A., Stieber J., Hes R. (2018). Patients at the Highest Risk for Reherniation Following Lumbar Discectomy in a Multicenter Randomized Controlled Trial. JB JS Open Access.

[17] Ramnarayan R., Chaurasia B. (2023). The post spinal surgery syndrome: A review. J. Craniovertebr. Junction Spine.

[18] McDonald C.L., Alsoof D., Glueck J., Osorio C., Stone B., McCluskey L., Diebo B.G., Daniels A.H., Basques B.A. (2023). Adjacent Segment Disease After Spinal Fusion. JBJS Rev..

[19] Barrett K.K., Fukunaga D., Rolfe K.W. (2021). Perioperative major neurologic deficits as a complication of spine surgery. Spinal Cord. Ser. Cases.

[20] Saadat N., Rezania K. (2021). Postoperative lumbar paraspinal compartment syndrome. BMJ Case Rep..

[21] Chaparro L.E., Smith S.A., Moore R.A., Wiffen P.J., Gilron I. (2013). Pharmacotherapy for the prevention of chronic pain after surgery in adults. Cochrane Database Syst. Rev..

[22] Palmisciano P., Balasubramanian K., Scalia G., Sagoo N.S., Haider A.S., Bin Alamer O., Chavda V., Chaurasia B., Deora H., Passanisi M. (2022). Posterior epidural intervertebral disc migration and sequestration: A systematic review. J. Clin. Neurosci..

[23] Al-Zamil M.K. (2022). Effectiveness of osteoelectroneurostimulation in treatment of patients with lumboishialgia. Vestnic Med. Stomatol. Inst..

[24] Rezasoltani Z., Ehyaie H., Mofrad R.K., Vashaei F., Mohtasham R., Najafi S. (2021). Granisetron vs. lidocaine injection to trigger points in the management of myofascial pain syndrome: A double-blind randomized clinical trial. Scand. J. Pain.

[25] Appasamy M., Lam C., Alm J., Chadwick A.L. (2022). Trigger Point Injections. Phys. Med. Rehabil. Clin. N. Am..

[26] Dernek B., Adiyeke L., Duymus T.M., Gokcedag A., Kesiktas F.N., Aksoy C. (2018). Efficacy of Trigger Point Injections in Patients with Lumbar Disc Hernia without Indication for Surgery. Asian Spine J..

[27] Yang X., Wei X., Mu Y., Li Q., Liu J. (2020). A review of the mechanism of the central analgesic effect of lidocaine. Medicine.

[28] Hong C.Z. (1994). Lidocaine injection versus dry needling to myofascial trigger point. The importance of the local twitch response. Am. J. Phys. Med. Rehabil..

[29] Al-Zamil M.K. (2022). Efficiency of direct electrotherapy peroneal and tibial nerves in the treatment of patients with lumbosacral post-discectomy syndrome. Vestnic Med. Stomatol. Inst..

[30] Al-Zamil M.K., Kulikova N.G., Vasilyeva E.S., Minenko I.A., Stepanovich M.A. (2021). Discogenic theory of lumbar osteochondrosis. A review of literature. Clin. Neurol..

[31] Zhuk Y.M., Al-Zamil M.K. (2021). Pain from Aristotle to Melzack and wall, literature review. Clin. Neurol..

[32] Heitler B. (2023). Primary Afferent Depolarization and the Gate Control Theory of Pain: A Tutorial Simulation. J. Undergrad. Neurosci. Educ..

[33] Hernández-Ortíz A.R., Ponce-Luceño R., Sáez-Sánchez C., García-Sánchez O., Fernández-de-Las-Peñas C., de-la-Llave-Rincón A.I. (2020). Changes in Muscle Tone, Function, and Pain in the Chronic Hemiparetic Shoulder after Dry Needling within or Outside Trigger Points in Stroke Patients: A Crossover Randomized Clinical Trial. Pain. Med..

[34] Xie G., Wang T., Tang X., Guo X., Xu Y., Deng L., Sun H., Ma Z., Ai Y., Jiang B. (2020). Acupoint Injection for Nonspecific Chronic Low Back Pain: A Systematic Review and Meta-Analysis of Randomized Controlled Studies. Evid. Based Complement. Alternat Med..

[35] Hammi C., Schroeder J.D., Yeung B. (2023). Trigger Point Injection.

[36] Bennett R. (2007). Myofascial pain syndromes and their evaluation. Best Pract. Res. Clin. Rheumatol..

[37] Dunning J., Butts R., Young I., Mourad F., Galante V., Bliton P., Tanner M., Fernández-de-Las-Peñas C. (2018). Periosteal Electrical Dry Needling as an Adjunct to Exercise and Manual Therapy for Knee Osteoarthritis: A Multicenter Randomized Clinical Trial. Clin. J. Pain.

[38] Mansur N. (2022). Comparative analysis between paravertebral and intraosseous blockades in treatment of patients with lumbar osteochondrosis related severe low back pain. Clin. Neurol..

[39] Han S.P., Han J.S. (2020). Acupuncture and Related Techniques for Pain Relief and Treatment of Heroin Addiction: Mechanisms and Clinical Application. Med. Acupunct..

[40] Hein G.N. (1918). Local Anesthesia, Infiltrative, Conductive and Intraosseous Methods. Dent. Regist..

[41] Sokov E.L., Arsiukhin N.A., Kornilova L.E., Nozdriukhina N.V. (2012). The complex treatment of the pain syndrome in diabetic distal polyneuropathy using intraosteal blockades. Zh. Nevrol. Psikhiatr. Im. SS Korsakova.

[42] Sokov E.L., Kornilova L.E., Filimonov V.A., Ganzhula P.A. (2009). Osteogenic factor in pathogenesis of vertebrogenic cardialgia. Klin. Med..

[43] Sokov E.L., Kornilova L.E., Filimonov V.A., Kliueva V.N. (2008). Effectiveness of intraosseous blockades in the treatment of spondylogenic disorders. Zh. Nevrol. Psikhiatr. Im. SS Korsakova.

[44] Shevelev O.A., Sokov E.L. (1993). The characteristics of the modulation of the afferent reactions during stimulation of the intraosseous receptors. Biull Eksp. Biol. Med..

[45] https://www.unboundmedicine.com/icd/view/ICD-10-CM/862551/all/M96_1___Postlaminectomy_syndrome__not_elsewhere_classified.

[46] Abou-Al-Shaar H., Adogwa O., Mehta A.I. (2018). Lumbar Spinal Stenosis: Objective Measurement Scales and Ambulatory Status. Asian Spine J..

[47] Talebian P., Golbakhsh M., Mirzashahi B., Zarei M., Rahimian A., Soleimani M. (2023). Validation and reliability of the Persian version of the Zurich Claudication Questionnaire in patients with lumbar spinal stenosis. N. Am. Spine Soc. J..

[48] Araki M., Nonoshita H., Kitano S., Shigematsu H., Tanaka M., Kawasaki S., Suga Y., Yamamoto Y., Tanaka Y. (2021). The critical cutoff point of the Zurich Claudication Questionnaire and the Japanese Orthopaedic Association score indicating locomotive syndrome in patients with lumbar spinal canal stenosis. J. Orthop. Sci..

[49] U.S. Department of Health and Human Services (2018). Physical Activity Guidelines for Americans.

[50] Rush A.J., South C.C., Jha M.K., Grannemann B.D., Trivedi M.H. (2019). Toward a very brief quality of life enjoyment and Satisfaction Questionnaire. J. Affect. Disord..

[51] Riendeau R.P., Sullivan J.L., Meterko M., Stolzmann K., Williamson A.K., Miller C.J., Kim B., Bauer M.S. (2018). Factor structure of the Q-LES-Q short form in an enrolled mental health clinic population. Qual. Life Res..

[52] Wong C.S., Wong S.H. (2012). A new look at trigger point injections. Anesthesiol. Res. Pract..

[53] Wan Q., Lin C., Li X., Zeng W., Ma C. (2015). MRI assessment of paraspinal muscles in patients with acute and chronic unilateral low back pain. Br. J. Radiol..

[54] (2005). Gray’s Anatomy: The Anatomical Basis of Clinical Practice.

[55] Dickson K.F., Stannard J., Schmidt A., Kregor P. (2007). Pelvic Ring Injuries. Surgical Treatment of Orthopaedic Trauma.

[56] Carayannopoulos N.L. (2017). The proximal origin of the gluteus maximus: A cadaveric study. J. Clin. Exp. Orthop..

[57] Lurie J.D., Tosteson A.N., Tosteson T.D., Carragee E., Carrino J.A., Kaiser J., Sequeiros R.T., Lecomte A.R., Grove M.R., Blood E.A. (2008). Reliability of readings of magnetic resonance imaging features of lumbar spinal stenosis. Spine.

[58] El Melhat A.M., Youssef A.S.A., Zebdawi M.R., Hafez M.A., Khalil L.H., Harrison D.E. (2024). Non-Surgical Approaches to the Management of Lumbar Disc Herniation Associated with Radiculopathy: A Narrative Review. J. Clin. Med..

[59] Patel V.B., Wasserman R., Imani F. (2015). Interventional Therapies for Chronic Low Back Pain: A Focused Review (Efficacy and Outcomes). Anesth. Pain. Med..

[60] Zhang X., Peng B., Zhao Z., Wu B., Ma Z., Wang G. (2023). Management of Post-lumbar-operation Back Pain using Myofascial Trigger Point Injection: A Retrospective Study. Iran. Red. Crescent Med. J..

[61] Khoshnazar S.S., Farpour H.R., Shahriarirad R. (2023). A comparison between effectiveness of gluteal trigger point and epidural steroid injection in lumbosacral canal stenosis patients: A randomized clinical trial. Br. J. Neurosurg..

[62] Kocak A.O., Ahiskalioglu A., Sengun E., Gur S.T.A., Akbas I. (2019). Comparison of intravenous NSAIDs and trigger point injection for low back pain in ED: A prospective randomized study. Am. J. Emerg. Med..

[63] Funk M.F., Frisina-Deyo A.J. (2020). Dry needling for spine related disorders: A scoping review. Chiropr. Man. Therap.

[64] Koppenhaver S.L., Walker M.J., Su J., McGowen J.M., Umlauf L., Harris K.D., Ross M.D. (2015). Changes in lumbar multifidus muscle function and nociceptive sensitivity in low back pain patient responders versus non-responders after dry needling treatment. Man. Ther..

[65] Sokov E.L., Kornilova L.E., Artiukov O.P. (2013). Intraosseous blocks in the treatment of symmetrical distal diabetic polyneuropathy. Ter. Arkh..

[66] Roldan C.J., Osuagwu U., Cardenas-Turanzas M., Huh B.K. (2020). Normal Saline Trigger Point Injections vs Conventional Active Drug Mix for Myofascial Pain Syndromes. Am. J. Emerg. Med..

[67] Suputtitada A., Chen C.P.C., Pongpirul K. (2024). Mechanical Needling with Sterile Water Versus Lidocaine Injection for Lumbar Spinal Stenosis. Global Spine J..

[68] Misirlioglu T.O., Akgun K., Palamar D., Erden M.G., Erbilir T. (2015). Piriformis syndrome: Comparison of the effectiveness of local anesthetic and corticosteroid injections: A double-blinded, randomized controlled study. Pain. Physician.

[69] Hasuo H., Matsuoka H., Matsuda Y., Fukunaga M. (2021). The Immediate Effect of Trigger Point Injection with Local Anesthetic Affects the Subsequent Course of Pain in Myofascial Pain Syndrome in Patients with Incurable Cancer by Setting Expectations as a Mediator. Front. Psychiatry.

[70] Peterfreund R.A., Vale W.W. (1986). Local anesthetics inhibit veratridine-induced secretion of calcitonin gene-related peptide (CGRP) from cultured rat trigeminal ganglion cells. Brain Res..

[71] Li Y.M., Wingrove D.E., Too H.P., Marnerakis M., Stimson E.R., Strichartz G.R., Maggio J.E. (1995). Local anesthetics inhibit substance P binding and evoked increases in intracellular Ca^2+^. Anesthesiology.

[72] Lapin G.A., Hochman B., Maximino J.R., Chadi G., Ferreira L.M. (2016). Effects of Lidocaine, Bupivacaine, and Ropivacaine on Calcitonin Gene-Related Peptide and Substance P Levels in the Incised Rat Skin. Adv. Skin. Wound Care.

[73] Hamaya C., Barr T., Strichartz G.R. (2018). Multiple Inhibitory Mechanisms of Lidocaine on Bradykinin Receptor Activity in Model Sensory Neurons. Reg. Anesth. Pain. Med..

[74] Weinschenk S., Weiss C., Benrath J., von Baehr V., Strowitzki T., Feißt M. (2022). Anti-Inflammatory Characteristics of Local Anesthetics: Inhibition of TNF-α Secretion of Lipopolysaccharide-Stimulated Leucocytes in Human Blood Samples. Int. J. Mol. Sci..

[75] Vinyes D., Muñoz-Sellart M., Fischer L. (2023). Therapeutic Use of Low-Dose Local Anesthetics in Pain, Inflammation, and Other Clinical Conditions: A Systematic Scoping Review. J. Clin. Med..

[76] Al-Zamil M.K., Kulikova N.G., Vasilyeva E.S., Minenko I.A., Stepanovich M.A. (2021). Application of Needle electromyography in the study of a patient with myotinic ayndrome after undergoing L5-S1 discectomy. Clin. Neurol..

[77] Lim T.K., Ma Y., Berger F., Litscher G. (2018). Acupuncture and Neural Mechanism in the Management of Low Back Pain—An Update. Medicines.

[78] Şengül M., Tekeli Şengül S. (2024). Efficacy of trigger point injection therapy in noncardiac chest pain: A randomized controlled trial. Turk. J. Phys. Med. Rehabil..

[79] Peterson E., Finkel J. (2021). Trigger point injections for axial back pain in adolescents. BMJ Case Rep..

[80] Hicks B.L., Lam J.C., Varacallo M. (2024). Piriformis Syndrome.

[81] Saeidian S.R., Pipelzadeh M.R., Rasras S., Zeinali M. (2014). Effect of trigger point injection on lumbosacral radiculopathy source. Anesth. Pain. Med..

[82] Moriwaki K., Shiroyama K., Yasuda M., Uesugi F. (2019). Reversible tactile hypoesthesia associated with myofascial trigger points: A pilot study on prevalence and clinical implications. Pain. Rep..

[83] Stepanovich M.A., Al-Zamil M., Kulikova N.G., Vaslyeva E.S., Minenko I.A. (2021). Segmental sensory deficits as a consequence of alleviation of neuropathic pain syndrome. J. Clin. Neurol..

[84] Al-Zamil M. (2016). Method of Forecasting Surface Sensitivity When Treating Diabetic Distal Polyneuropathy of Lower Extremities with Pain Syndrome—R U 2593227 C 1. Bull. Invent. Util. Models.

[85] Egloff N., Sabbioni M.E., Salathé C., Wiest R., Juengling F.D. (2009). Nondermatomal somatosensory deficits in patients with chronic pain disorder: Clinical findings and hypometabolic pattern in FDG-PET. Pain.

[86] Mailis-Gagnon A., Nicholson K. (2010). Nondermatomal somatosensory deficits: Overview of unexplainable negative sensory phenomena in chronic pain patients. Curr. Opin. Anaesthesiol..

[87] Landmann G., Dumat W., Egloff N., Gantenbein A.R., Matter S., Pirotta R., Sándor P.S., Schleinzer W., Seifert B., Sprott H. (2017). Bilateral Sensory Changes and High Burden of Disease in Patients with Chronic Pain and Unilateral Nondermatomal Somatosensory Deficits: A Quantitative Sensory Testing and Clinical Study. Clin. J. Pain.

[88] Matesanz L., Hausheer A.C., Baskozos G., Bennett D.L.H., Schmid A.B. (2021). Somatosensory and psychological phenotypes associated with neuropathic pain in entrapment neuropathy. Pain.

[89] Rehm S., Sachau J., Hellriegel J., Forstenpointner J., Børsting Jacobsen H., Harten P., Gierthmühlen J., Baron R. (2021). Pain matters for central sensitization: Sensory and psychological parameters in patients with fibromyalgia syndrome. Pain. Rep..

[90] Knight M.T., Jago I., Norris C., Midwinter L., Boynes C. (2014). Transforaminal endoscopic lumbar decompression & foraminoplasty: A 10 year prospective survivability outcome study of the treatment of foraminal stenosis and failed back surgery. Int. J. Spine Surg..

[91] McGrath L.B., Madhavan K., Chieng L.O., Wang M.Y., Hofstetter C.P. (2016). Early experience with endoscopic revision of lumbar spinal fusions. Neurosurg. Focus.

[92] McIlroy S., Jadhakhan F., Bell D., Rushton A. (2021). Prediction of walking ability following posterior decompression for lumbar spinal stenosis. Eur. Spine J..

[93] Sikdar S., Ortiz R., Gebreab T., Gerber L.H., Shah J.P. (2010). Understanding the vascular environment of myofascial trigger points using ultrasonic imaging and computational modeling. Annu. Int. Conf. IEEE Eng. Med. Biol. Soc..

[94] Dorigo B., Bartoli V., Grisillo D., Beconi D. (1979). Fibrositic myofascial pain in intermittent claudication. Effect of anesthetic block of trigger points on exercise tolerance. Pain.

[95] Markman J.D., Gewandter J.S., Frazer M.E., Pittman C., Cai X., Patel K.V., Jahromi B.S., Dworkin R.H., Burke L.B., Farrar J.T. (2015). Evaluation of outcome measures for neurogenic claudication: A patient-centered approach. Neurology.

[96] Nurmukhametov R., Medetbek A., Ramirez M.E., Afsar A., Sharif S., Montemurro N. (2023). Factors affecting return to work following endoscopic lumbar foraminal stenosis surgery: A single-center series. Surg. Neurol. Int..

[97] Steverink J.G., Oostinga D., van Tol F.R., van Rijen M.H.P., Mackaaij C., Verlinde-Schellekens S.A.M.W., Oosterman B.J., Van Wijck A.J.M., Roeling T.A.P., Verlaan J.J. (2021). Sensory Innervation of Human Bone: An Immunohistochemical Study to Further Understand Bone Pain. J. Pain.

[98] Sokov E.L., Shevelev O.A. (1994). The osteogenic mechanism of vertebrogenic radiculopathies. Zh. Nevrol. Psikhiatr. Im. SS Korsakova.

[99] Mao B.Y. (1993). The change of intraosseous pressure in the femur and tibia near knee and knee pain. Zhonghua Wai Ke Za Zhi.

[100] Andrew C., Bassett Robert L., Becker O. (1962). Generation of Electric Potentials by Bone in Response to Mechanical Stress. Science.

[101] Yankovskis G., Beldava I., Liviņa B. (2000). Osteoreflectory treatment of alcohol abstinence syndrome and craving for alcohol in patients with alcoholism. Acupunct. Electrother. Res..

[102] Albagieh H., Aloyouny A., Alshehri N., Alsammahi N., Almutrafi D., Hadlaq E. (2020). Efficacy of lidocaine versus mepivacaine in the management of myofascial pain. Saudi Pharm. J..

[103] Robbins M.S., Kuruvilla D., Blumenfeld A., Charleston L., Sorrell M., Robertson C.E., Grosberg B.M., Bender S.D., Napchan U., Ashkenazi A. (2014). Peripheral Nerve Blocks and Other Interventional Procedures Special Interest Section of the American Headache Society. Trigger point injections for headache disorders: Expert consensus methodology and narrative review. Headache.

[104] Zaralidou A.T., Amaniti E.N., Maidatsi P.G., Gorgias N.K., Vasilakos D.F. (2007). Comparison between newer local anesthetics for myofascial pain syndrome management. Methods Find. Exp. Clin. Pharmacol..

[105] Tschopp K.P., Gysin C. (1996). Local injection therapy in 107 patients with myofascial pain syndrome of the head and neck. ORL J. Otorhinolaryngol. Relat. Spec..

